# Amphiphilic Emulgels Loaded with Pomegranate Carbon Dots and Rosemary Oil for Metabolic pH Monitoring

**DOI:** 10.3390/gels12080696

**Published:** 2026-08-04

**Authors:** Hebat-Allah S. Tohamy, Ilaria Cacciotti

**Affiliations:** 1 Cellulose and Paper Department, National Research Centre, 33 El Bohouth Str., Dokki, Giza P.O. Box 12622, Egypt; 2 Department of Engineering, INSTM RU, University of Rome “Niccolò Cusano”, 00166 Rome, Italy

**Keywords:** pomegranate-derived carbon dots, heat-map, polyelectrolyte, rosemary essential oil, amphiphilic emulgel, food spoilage sensing, metabolic pH signatures, nanocomposite complex stabilization

## Abstract

The development of sustainable, smart food packaging materials that simultaneously provide antimicrobial protection and real-time monitoring of food quality is a critical frontier in food safety. This study reports the fabrication of a multifunctional amphiphilic emulgel designed for the detection of pathogen-induced metabolic pH changes in food systems. The system utilizes Pomegranate-derived nitrogen-doped quasi-spherical carbon dots (QS-CDs) as fluorescent nanoprobes and Rosemary Essential Oil (REO) as a natural antimicrobial agent, both encapsulated within a polyelectrolyte complex of chitosan and sugarcane bagasse-derived carboxymethyl cellulose (CMC). A low degree of substitution (DS = 0.4) was specifically engineered for the CMC to ensure an amphiphilic character, enabling nanocomposite complex stabilization of the REO droplets without synthetic surfactants. Structural characterization via Transmission Electron Microscopy (TEM) revealed well-dispersed QS-CDs (4.71–6.62 nm) and stable oil droplets (~605.49 nm) anchored within a zipped polymer network. Thermal analysis (TGA/DSC) using the Coats–Redfern model revealed a significant synergistic effect: the smart-emulgel exhibits a distinct two-stage degradation profile, with the high-temperature stage requiring an activation energy (E_a_) of 95.19 kJ/mol, a substantial increase over the corresponding stage in the CD-emulgel baseline (18.69 kJ/mol). This enhanced stability is complemented by a slight increase in crystallinity (X_c_ from 0.11 to 0.14). While the smart-emulgel remains predominantly amorphous, this shift suggests that the integration of REO and QS-CDs into the polymer network promotes the formation of localized, more ordered domains, contributing to a more robust and structurally integrated matrix. The emulgel demonstrated a dual-mode optical response (colorimetric and fluorometric) sensitive to the metabolic byproducts (e.g., organic acids, amines, other alkaline compounds) produced by *Escherichia coli* and *Staphylococcus aureus*. These findings were corroborated by Density Functional Theory (DFT) calculations, which confirmed the thermodynamic stability and optimized electronic energy gaps for pH-responsive sensing. This research provides a green, high-performance platform for the real-time monitoring of food freshness and the prevention of foodborne illnesses.

## 1. Introduction

Transitioning toward a circular bioeconomy is a critical imperative for ensuring global food security [1,2,3,4]. This concept has evolved from a theoretical framework into a fundamental necessity for global food security. Despite rapid advances in food technology, current monitoring systems remain largely dependent on a reliance on synthetic indicators and fragile delivery platforms. Many conventional smart labels rely on synthetic pH dyes or heavy-metal-based quantum dots which, although research addresses this imperative by repurposing discarded biomass into high-value functional materials, establishing a sustainable bridge between waste valorization and advanced food-sensing technologies [5,6,7,8,9], many conventional smart labels utilize synthetic pH dyes or heavy-metal-based quantum dots. While these materials have been successfully validated for specific food-contact applications and can be safe when properly immobilized, concerns persist regarding potential chemical leaching and toxicity. Conversely, while biomass-derived nanoprobes offer a promising sustainable alternative, they are not inherently biocompatible by virtue of their origin [10,11,12]. Furthermore, while the industry has looked toward essential oils as a clean-label antimicrobial alternative, their integration into food matrices is notoriously difficult and remains challenging. In the absence of suitable carriers, these volatile compounds suffer from rapid evaporation, poor solubility, and phase separation. Consequently, they tend to be accumulated at the surface rather than remaining homogeneously dispersed to fight pathogens, thereby reducing antimicrobial efficacy [11,13,14,15,16]. This inherent instability typically results in short-lived and inconsistent performance. There is, therefore, an urgent need for advanced encapsulation strategies that can simultaneously stabilize these volatile bioactives and replace toxic synthetic sensing elements with biocompatible, sustainable, green nanoprobes.

The challenge lies in designing a matrix that combines high structural stability against oil droplet coalescence with the sensitivity required to generate a clear and timely response to changes relevant to the pH changes before visible spoilage becomes apparent, if experimentally validated.

In this framework, agro-industrial wastes such as pomegranate peels and sugarcane bagasse are repurposed as cellulose-rich agro-industrial residue precursors, bridging the gap between environmental stewardship and nanotechnological innovation [17,18]. The pomegranate exocarp provides carbon- and nitrogen-rich sources for the rapid microwave-assisted green synthesis of quasi-spherical carbon dots (QS-CDs), while the sugarcane bagasse-derived cellulose is chemically modified and engineered into low-degree-of-substitution carboxymethyl cellulose (CMC) [19,20,21,22]. This specific functionalization transforms CMC into an amphiphilic polymer, capable of stabilizing essential oils without the need for synthetic petroleum-based surfactants [23,24,25].

By integrating these upcycled biomass-derived carbon sources into a polyelectrolyte matrix, the agro-industrial byproducts are converted into active sensing and antimicrobial elements for the real-time detection of microbial activity. Building on this sustainable foundation, the emulgel evolves from a passive carrier to an active biological system through the incorporation of Rosemary essential oil (REO) and carbon dots. REO acts as the primary antimicrobial constituent within the emulgel architecture, effectively disrupting the phospholipid bilayers of the bacterial cell membranes, thereby inhibiting microbial proliferation. This approach offers a bio-based natural alternative to synthetic preservatives for sustainable and active food packaging applications [26,27,28,29,30]. However, protective function alone is insufficient, and smart functionality of the system is required in order to communicate environmental changes.

The integration of pomegranate-derived QS-CDs as nanoprobes within the polyelectrolyte matrix establishes a continuous optical sensing framework, enabling high-sensitivity monitoring of environmental variations through their consistent fluorometric response. These nanoprobes act as molecular transducers that detect the metabolic signatures of spoilage, specifically the localized acidification under carbohydrate-rich conditions associated with the proliferation of *Escherichia coli* (*E. coli*) or *Staphylococcus aureus* (*S. aureus*) [31,32]. As bacteria consume nutrients and release acidic byproducts, the protonation of nitrogen-rich surface groups on the carbon dots induces rapid and reversible changes in both colorimetric and fluorescence signals [33,34]. This dual-mode response transforms the packaging system into an intelligent platform, providing a real-time, naked-eye warning of contamination while simultaneously providing antimicrobial features.

Crucial to the material’s structural integrity is the nanocomposite complex stabilization mechanism, which avoids the use of conventional synthetic surfactants [35,36,37]. This is achieved by engineering the CMC to an optimal “sweet spot” degree of substitution (DS) of 0.4. At this precise level, the CMC is no longer just a water-soluble polymer; it becomes an amphiphilic mediator [34]. Its hydrophobic segments firmly anchor into the rosemary essential oil droplets, while its hydrophilic carboxylate groups remain in the aqueous phase, forming a stable physical barrier at the oil–water interface, which prevents the oil droplets’ coalescence and phase separation. Central to the diagnostic power of this emulgel is its ability to decode the metabolic pH signatures generated by invading microorganisms. Traditional sensors often provide a static measurement of the environment, but bacteria can alter their local microenvironment through metabolic activity, pH changes, and the release of extracellular products [38,39,40].

As these bacteria consume nutrients within a food matrix, they engage in anaerobic fermentation and metabolic processes that release a consistent stream of organic acids and chemical footprints [41,42,43]. The developed composite acts as an active, stimuli-responsive interface rather than a passive barrier. It exhibits high analytical sensitivity to subtle fluctuations in the chemical environment, specifically transducing localized pH variations into measurable optical outputs. The nitrogen-doped surfaces of the quasi-spherical carbon dots are specifically tuned to interact with these metabolic byproducts. When the concentration of acidic metabolites reaches a critical threshold, it triggers a protonation event across the emulgel’s matrix, resulting in a quantifiable optical shift [31,44]. By focusing on these signatures, we move beyond simple pH detection and enter the realm of real-time biological surveillance, where there is a time-dependent optical record of chemical changes associated with microbial metabolism. This allows for the detection of contamination long before macroscopic signs of spoilage, such as odor or texture changes, become apparent to the consumer.

The stability is further reinforced through an electrostatic zipping mechanism [45,46]. When the cationic chitosan with its positively charged amine groups is introduced to the anionic, CMC-coated oil droplets, a spontaneous polyelectrolyte complexation occurs [44,47,48]. This zips the droplets into a rigid, three-dimensional network, effectively trapping both the antimicrobial oil and the fluorescent carbon dots in a stable matrix. This architecture ensures that the smart components remain exactly at the interface where they can immediately detect and combat bacterial growth. In light of these challenges, the scope of this work encompasses the design, fabrication, and systematic characterization of a multifunctional smart-emulgel that combines antimicrobial activity with pH-responsive optical monitoring. To bridge the gap between experimental observation and molecular theory, we employed a multi-scale characterization approach. To bridge the gap between experimental observation and molecular theory, we employed a multi-scale characterization approach. The morphological architecture and the precise nanometric integration of the QS-CDs were evaluated using Transmission Electron Microscopy (TEM). The thermal resilience and the unique synergistic interaction of the polyelectrolyte matrix were assessed through Thermogravimetric Analysis (TGA) utilizing the Coats–Redfern model. Finally, the study provides theoretical insights into the electronic structure, frontier molecular orbitals, and energy gaps of the system using Density Functional Theory (DFT) calculations. Collectively, these techniques validate the smart-emulgel as a high-performance, integrated multifunctional platform capable of providing a green, real-time solution for global food safety monitoring.

## 2. Results and Discussion

### 2.1. Chemical Analysis of the Prepared Precursors with Theoretical DFT Calculations

**Figure 1:**
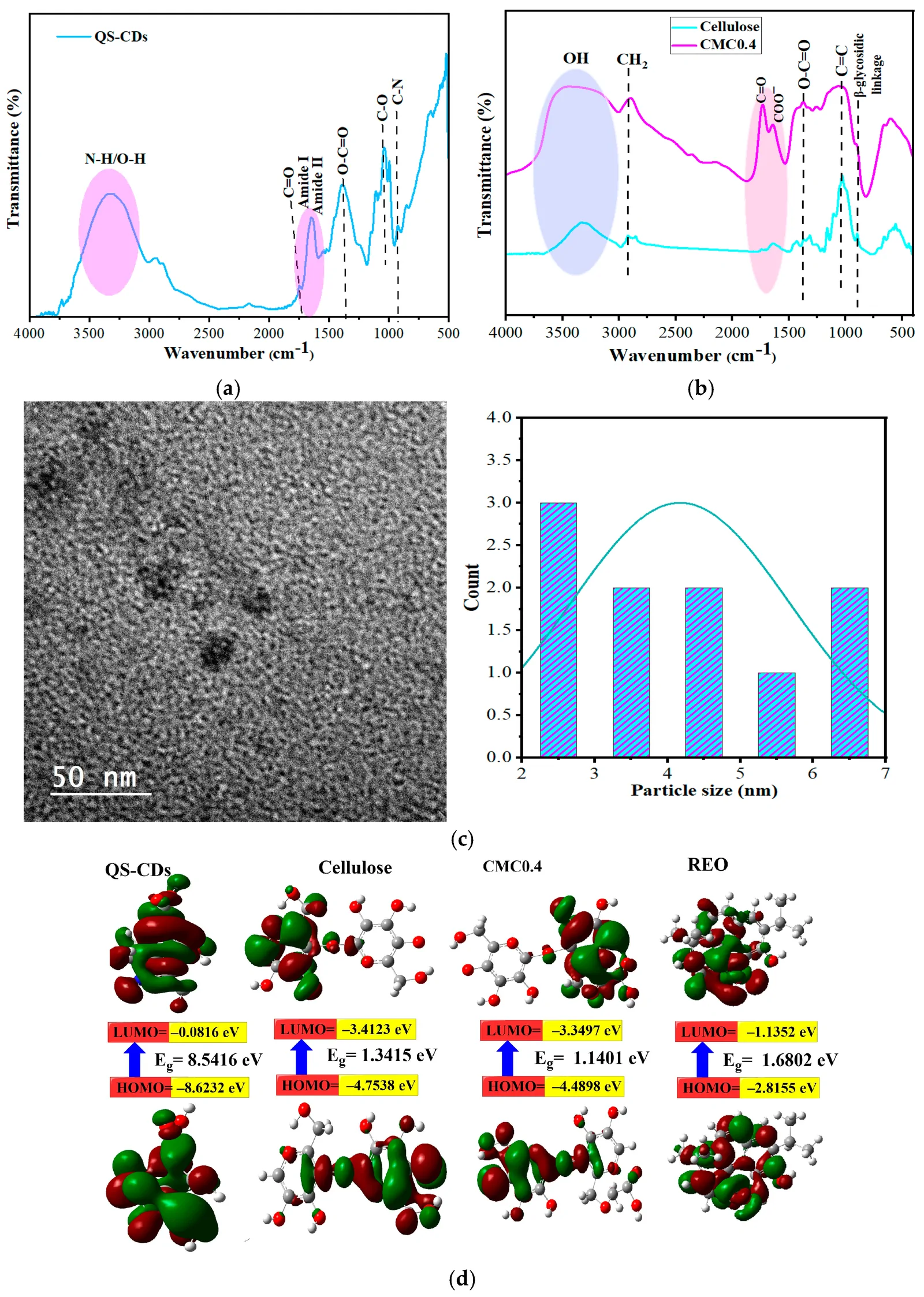
(**a**) FTIR of QS-CDs, (**b**) FTIR of cellulose and CMC0.4, (**c**) TEM of QS-CDs with particle size distribution histogram, and (**d**) DFT of QS-CDs, cellulose, CMC0.4, and REO oil.

The FTIR spectrum of QS-CDs (Figure 1a) shows peaks at 3316 cm^−1^ (N–H/O–H), 1749 cm^−1^ (protonated carboxylic acid groups (–COOH)), 1646 cm^−1^ (Amide I), 1542 cm^−1^ (Amide II), 1392 cm^−1^ (O–C=O), 1039 cm^−1^ (C–O), and 927 cm^−1^ (C–N), reflecting the nitrogen-doped surface states of the carbon dots. These assignments are consistent with the formation of a stable, carbonized nanostructure, as also confirmed by TEM analysis (Figure 1c) [19,44,49]. The relative absorbance of the C–N band (A_(C–N)_/A_CH_) = 1.10 was used as a semi-quantitative metric to monitor the extent of nitrogen doping in the carbonized nanostructures.

The chemical transformation of sugarcane bagasse-derived cellulose into amphiphilic CMC (DS = 0.4) was also confirmed through FTIR spectroscopy. The raw cellulose exhibited characteristic vibrational bands at 3462 cm^−1^ (O–H stretching), 2882 cm^−1^ (C–H stretching), 1650 cm^−1^ (O–H bending of adsorbed water), 1375 cm^−1^ (CH_2_ bending), and 1040 cm^−1^, ascribed to the skeletal C–O–C stretching vibrations of the pyranose ring characteristic of the cellulosic backbone [31,34]. Distinctly, the presence of the β-glycosidic linkages was confirmed by the well-resolved band at 896 cm^−1^ for raw cellulose. Following functionalization into Na-CMC, this specific band shifted to 901 cm^−1^, indicating successful carboxymethylation and the resulting modification of the cellulose backbone vibrations. In the FTIR spectrum of CMC (DS = 0.4) the main cellulose peaks were retained, while new absorption bands at 1733 and 1643 cm^−1^ were detected. The band at 1643 cm^−1^ corresponds to the asymmetric stretching of deprotonated carboxylate (–COO^−^) groups, whereas the sharp peak at 1733 cm^−1^ is assigned to the carbonyl (C=O) stretching of protonated carboxylic acid groups (–COOH) (Figure 1b). The presence of this un-ionized carbonyl state provides direct evidence of a chemically dynamic system capable of undergoing rapid structural and optical transitions when exposed to the acidic metabolic byproducts of bacterial proliferation.

The emergence of these bands suggests the successful grafting of carboxymethyl moieties onto the cellulosic framework via the chloroacetic acid treatment. In particular, the peak at 1733 cm^−1^ plays a key role in pH responsiveness: under neutral conditions, carboxylate groups remain deprotonated, maintaining the stability of the polyelectrolyte network, whereas, under acidic conditions, due to the accumulation of acidic metabolic byproducts from bacterial proliferation, they undergo protonation, inducing contraction and subsequent optical shifts observed in the final material. The high degree of correlation between the potentiometric titration data and the FTIR absorbance ratio method (both yielding DS of 0.4) represents a significant methodological validation. This convergence of independent analytical techniques provides high confidence in the accuracy of the molecular structure and composition of the synthesized polymer. A DS of 0.4 is identified as the optimal nanocomposite complex stabilization. Unlike high-DS CMC (DS typically > 0.7), which is fully water-soluble and tends to solubilize into the bulk aqueous phase, the DS 0.4 CMC retains amphiphilic character. This specific level of substitution renders the polymer just insoluble enough to selectively migrate to the oil–water interface, stabilizing the REO/water interface, while remaining hydrophilic enough to ensure a stable, homogenous dispersion within the aqueous chitosan matrix.

The QS-CDs morphology was investigated using Transmission Electron Microscopy (TEM), revealing well-dispersed quasi-spherical nanoparticles (Figure 1c). As shown in the micrographs, the QS-CDs exhibit a narrow size distribution ranging from 4.71 to 6.62 nm. This ultra-small size is fundamental for the emulgel’s high performance, as the high surface-to-volume ratio maximizes the density of nitrogen-rich functional groups available for interaction with the polymer matrix. These nanometric dimensions allow the QS-CDs to seamlessly localize at the oil–water interface or within the interstitial spaces of the CMC/chitosan network without compromising the gel’s structural integrity. Furthermore, the particle size consistently staying below 10 nm confirms that the observed photoluminescence originates from quantum confinement and surface state transitions, which are essential for the sensitive dual-mode detection of bacterial metabolic activity in both the CD-emulgel and smart-emulgel systems.

Density Functional Theory (DFT) provides a molecular-level understanding of the electronic interactions governing the stability and reactivity of the QS-CDs, cellulose, and CMC within the emulgel matrix (Figure 1d, Table 1). The frontier molecular orbital analysis reveals that the QS-CDs possess the highest energy gap (E_g_ = 8.5416 eV), compared to cellulose (E_g_ = 1.3415 eV) and CMC (E_g_ = 1.1401 eV). This wider bandgap in the carbon dots highlights their superior electronic stability and explains their role as effective fluorescent probes [32,33]. In contrast, the significantly lower E_g_ values for cellulose and CMC suggest higher electronic reactivity, facilitating carboxymethylation and subsequent polyelectrolyte complexation [17,18].

The transition from raw cellulose to functionalized CMC is further elucidated by the total energy (E_T_) and dipole moment (µ) values. The total energy decreases from −1208.88 au in cellulose to −1434.55 au in CMC, indicating that the carboxymethylation process results in a thermodynamically more stable structure [32]. Simultaneously, the µ remains high in both cellulose (12.63 Debye) and CMC (11.14 Debye), confirming the strong molecular polarity, essential for maintaining an amphiphilic character. This high polarity is instrumental in the nanocomposite complex stabilization mechanism, as it ensures strong affinity between the polymer chains and the aqueous phase. Furthermore, the chemical hardness and softness parameters provide a clear picture of the system’s kinetic stability and chemical reactivity. The QS-CDs exhibited a high hardness value of 4.2708 eV, indicating a robust electronic configuration and significant resistance to changes in their electron cloud. This electronic rigidity suggests that the carbonized core of the dots remains stable during the emulgel fabrication process. In contrast, the CMC showed a significantly lower hardness value (0.5700 eV). This lower energy barrier for electronic perturbation in the CMC framework is beneficial for the polyelectrolyte complexation process, as it facilitates the electrostatic interactions and hydrogen bonding required to anchor the QS-CDs and stabilize the essential oil droplets within the zipped matrix. To further understand the driving forces behind the assembly of the smart-emulgel, the electrophilicity index (ω) was analyzed. The CMC exhibited a remarkably high electrophilicity index (13.4760 eV), whereas the QS-CDs showed a significantly lower value (2.2178 eV). This suggests that the CMC backbone acts as a strong electron-accepting matrix within the composite, promoting synergistic interactions with the electron-rich nitrogen-doped surface groups of the QS-CDs and the chitosan amine groups. This facilitates the formation of a robust, coordinated sensing network, ensuring that the fluorescent nanoprobes are firmly immobilized and electronically coupled to the polymer matrix for sensitive pH-responsive signaling. Collectively, these DFT parameters validate the electronic compatibility of all components, proving that the system is optimally designed for stable assembly and highly sensitive pH-responsive optical sensing.

### 2.2. Morphological Evolution of the Emulgels with Stability, DFT Calculations and Comparative Antimicrobial Efficacy Against Food Pathogens

The microstructural architecture and component integration of the three emulgel formulations were elucidated through TEM, revealing distinct morphological transitions as the system complexity increases. For the REO-emulgel, the micrographs display the successful formation of a stable emulsion system within the polyelectrolyte framework, characterized by well-defined spherical oil droplets, with some droplets exhibiting diameters in the sub-micron range (approximately 600 nm), effectively embedded within the cross-linked chitosan-CMC network.

**Figure 2:**
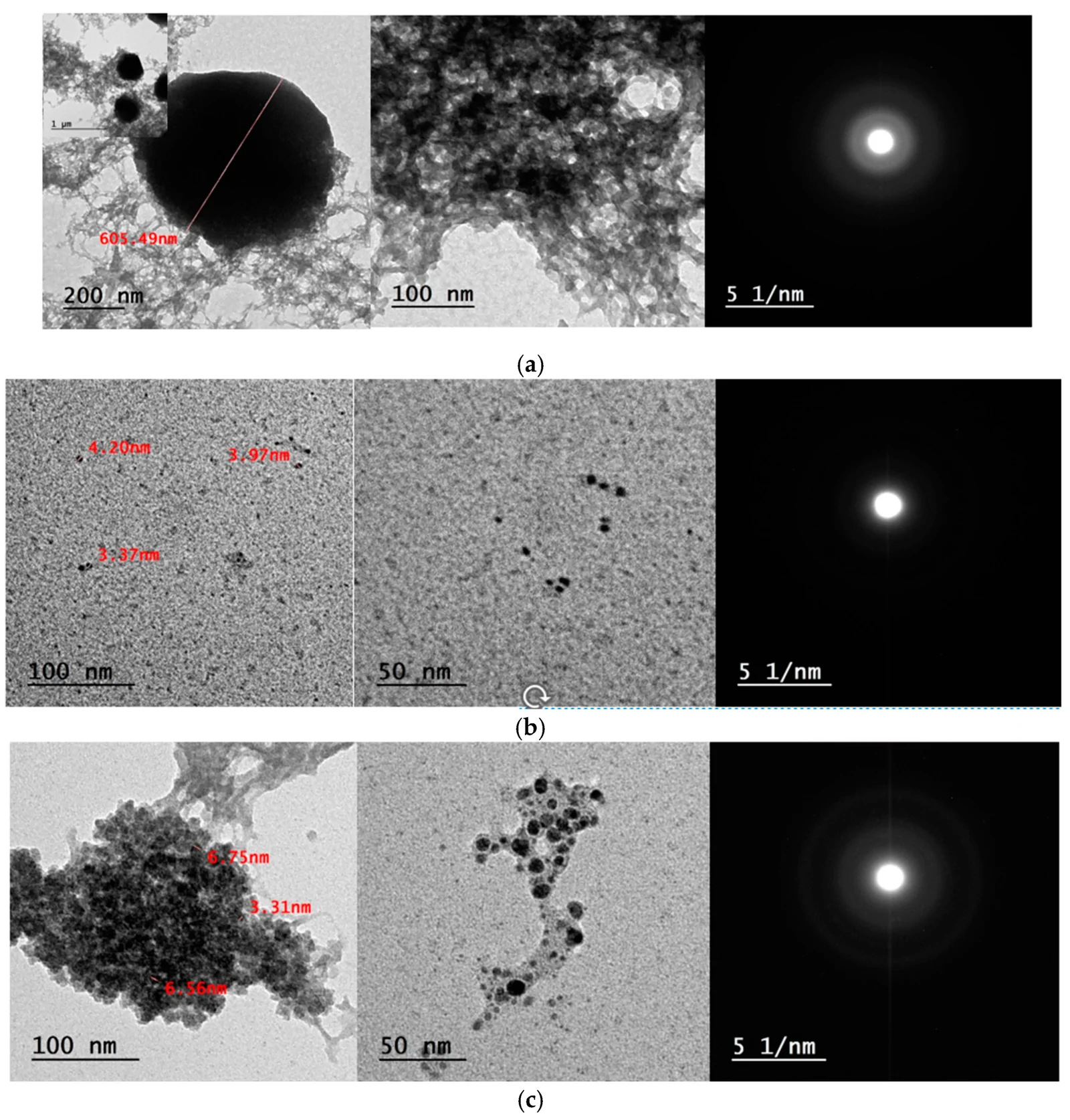

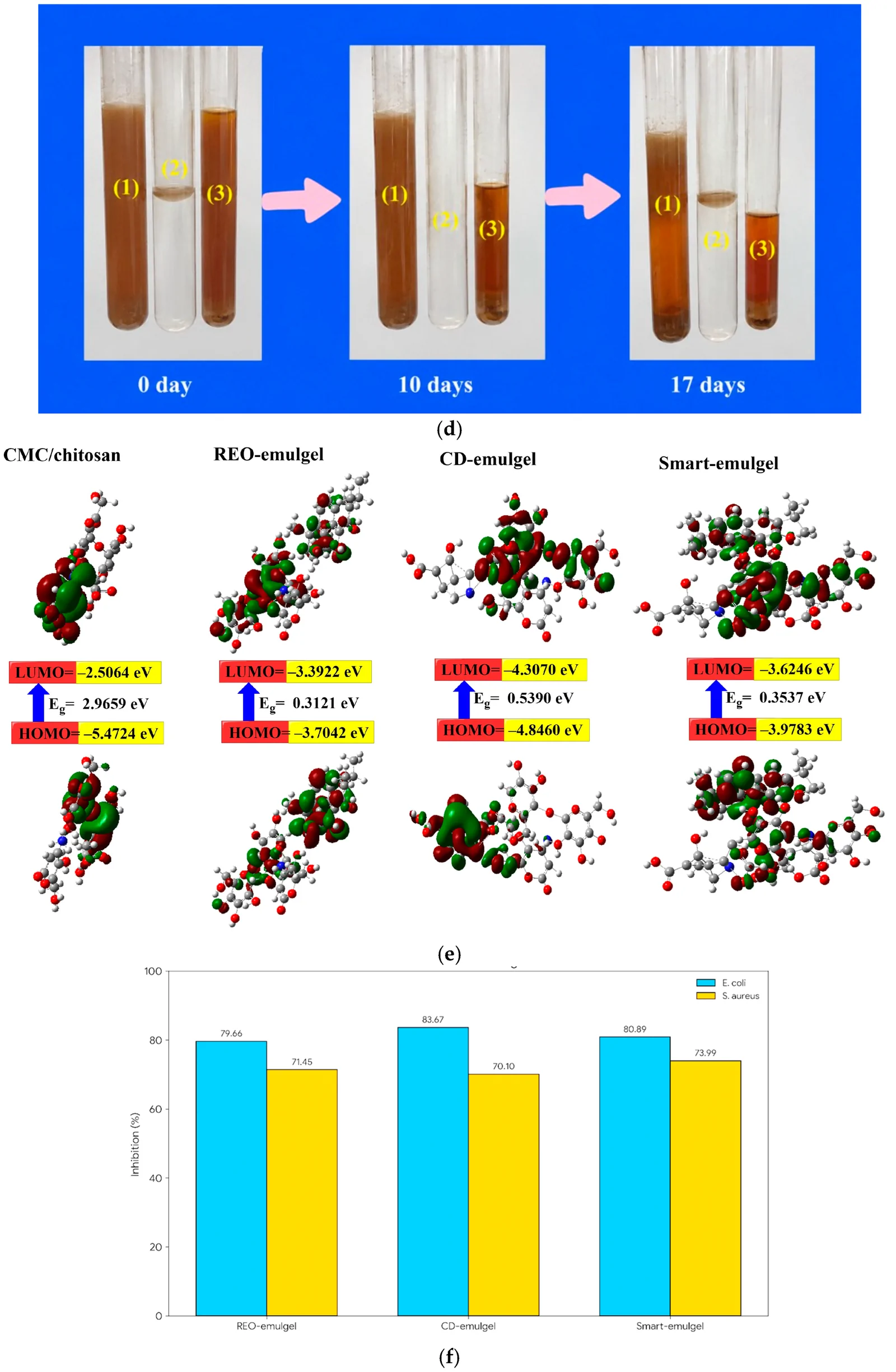
TEM analysis with SAED for (**a**) REO-emulgel, (**b**) CD-emulgel, & (**c**) Smart-emulgel, (**d**) Visual observation for the REO-emulgel (denoted as 2), CD-emulgel (denoted as 1), & Smart-emulgel (denoted as 3) over 17 days, (**e**) DFT calculations for REO-emulgel, CD-emulgel, & Smart-emulgel, and (**f**) Antimicrobial activity for CMC/chitosan, REO-emulgel, CD-emulgel, & Smart-emulgel.

This suggests that the DS 0.4 CMC successfully acts as an amphiphilic stabilizer, forming a film that prevents droplet coalescence. To evaluate the emulsion homogeneity and stability, the droplet size distribution was analyzed at multiple scales. The consistency between the nanometric encapsulation, evidenced in TEM analysis, and the long-term macroscopic stability suggests a highly controlled droplet size distribution, facilitated by the electrostatic zipping of the CMC-chitosan–QS-CDs framework which acts as a rigid 3D scaffold. The corresponding SAED pattern displays diffuse halo-like rings without sharp diffraction spots, suggesting a fully amorphous structure. This experimental evidence indicates that the polyelectrolyte complexation between chitosan and CMC generates a disordered polymeric matrix that securely encapsulates the liquid oil phase without inducing any long-range crystalline order (Figure 2a). Upon the incorporation of the QS-CDs in the CD-emulgel, well dispersed quasi-spherical QS-CDs with a narrow size distribution (3.37–4.20 nm) were evidenced in the TEM analysis (Figure 2b). The uniform dispersion highlights the anti-aggregation efficiency of the chitosan–CMC matrix which provides effective spatial confinement of the dots within the gel interstitial spaces, preventing nanoparticles clustering. This high degree of dispersion is critical for the emulgel’s optical performance, ensuring that the nitrogen-rich surface groups of the dots remain accessible for molecular sensing and photoluminescent signaling. The Selected Area Electron Diffraction (SAED) pattern for this formulation shows slightly more defined diffuse rings than in the REO-emulgel, suggesting that the presence of the carbon dots may introduce subtle electronic scattering. This supports that the QS-CDs are well-dispersed and integrated into the polymer matrix without causing large-scale aggregation. The overall lack of sharp rings suggests that the QS-CDs are well integrated at the molecular level, preserving the flexibility and transparency of the emulgel carrier (Figure 2b). The smart-emulgel represents the most structurally complex system, combining the structural features of both the oil phase and the fluorescent nanoprobes. It exhibits a synergistic triple-network architecture in which QS-CDs (3.31–6.75 nm) form a dense nanocomposite shell. This suggests interfacial nanocomposite complex stabilization, where QS-CDs and amphiphilic CMC (DS = 0.4) cooperate to form a rigid, solid-like barrier around the oil phase, bolstered by electrostatic zipping [50].

The TEM micrographs reveal a synergistic triple-network architecture where the QS-CDs reinforce the CMC/chitosan complex surrounding the REO droplets. This dense accumulation of nanostructures at the interface suggests that the QS-CDs contribute to the structural integrity of the emulgel, acting as reinforcing agents that strengthen the shell around the droplets. This triple-layer stabilization comprising CMC, chitosan, and QS-CDs creates a robust, dense protective layer around the REO core. In the final smart-emulgel formulation, the incorporation of nitrogen-doped QS-CDs serves a dual purpose: providing chromatic sensing capabilities. TEM analysis of the smart-emulgel network (Figure 2c) reveals a significantly higher electron density at the oil–water interface compared to the REO-emulgel template. This increased electron density is attributed to the adsorption of QS-CD clusters, which act as nanometric anchoring elements within the nanocomposite complex. This transition from a simple polyelectrolyte matrix to a dense nanocomposite shell explains the macroscopic stability of the system, which remained resistant to phase separation throughout all the storage period. The structural order of the fabricated emulgels was probed via SAED to understand the phase state of the matrices. The corresponding SAED patterns for the REO-emulgel (Figure 2a) and the CD-emulgel (Figure 2b) display diffuse halo-like rings without distinct Bragg reflections, suggesting the absence of long-range crystalline order. Indeed, this experimental finding confirmed a predominantly amorphous nature of the investigated systems, indicating that the electrostatic cross-linking between the cationic chitosan and the modified sugarcane bagasse CMC results in a highly disordered polymeric network. Structurally, this amorphous configuration implies that the matrix forms a random, glassy 3D mesh.

Rather than segregating into separate crystalline domains, the liquid active components (REO) and the nanoprobes are molecularly accommodated within the interstitial spaces of the polymer chains. In the final smart-emulgel formulation (Figure 2c), the SAED profile retains its characteristic diffuse halo, although a slight increase in ring definition is observable, corresponding to a minor increase in the calculated crystallinity degree (X*_c_* from 0.11 to 0.14). Crucially, the preservation of a largely amorphous matrix is highly advantageous for the smart-emulgel’s dual-mode functionality. The disordered polymeric architecture eliminates rigid crystalline boundaries, thereby facilitating the rapid and unrestricted diffusion of the volatile antimicrobial terpenes toward target pathogens. Furthermore, it ensures that the nitrogen-doped surface states of the encapsulated QS-CDs remain highly exposed and electronically unhindered, allowing for immediate protonation and an instant optical response when triggered by the acidic metabolic signatures of proliferating bacteria.

The long-term visual stability of the fabricated systems was evaluated through visual observation over a 17-day storage period, as illustrated in the provided physical samples. The REO-emulgel (2) and CD-emulgel (1) exhibited distinct phase behaviors compared to the integrated smart-emulgel (3).

To validate the visual observation over time which indicates physical storage stability or kinetic stability of the fabricated systems beyond localized microscopic observations, a macroscopic storage stability study was conducted via systematic visual inspection at room temperature (25 °C) across a 17-day tracking period.

As illustrated by the chronological physical profiles in Figure 2d, the individual control systems exhibited pronounced phase instabilities during storage, in contrast to the superior performance of the smart-emulgel formulation. In the baseline REO-emulgel (Sample 2), a severe phase separation became visually evident by days 10 and 17, characterized by extensive creaming and the formation of a highly transparent aqueous layer due to the upward migration of unanchored oil droplets.

This rapid destabilization indicates that, although high-shear homogenization initially generated sub-micron droplets such as the representative droplet with a diameter of 605.49 nm observed by TEM, the binary polymer template lacked sufficient structural rigidity to prevent droplet coalescence and subsequent flotation over time. Similarly, the CD-emulgel (Sample 1) exhibited noticeable localized sedimentation and particle precipitation at the bottom of the tube by day 17, suggesting that the nanoprobes require a co-encapsulated core to remain homogeneously dispersed within the polymer network. Conversely, the optimized smart-emulgel (Sample 3) demonstrated remarkable macroscopic stability throughout the entire 17-day observation period, maintaining a completely homogeneous, unified deep-amber appearance without detectable creaming, oil leaching, or bulk phase separation. This excellent physical consistency over time suggests that the single-droplet nanometric confinement observed by TEM translates into a macroscopically stable colloidal system. The persistent uniformity of the smart-emulgel supports the inclusion of nitrogen-doped QS-CDs alongside the DS 0.4 CMC and chitosan generates a tightly interconnected 3D viscoelastic network. This multi-component architecture provides immobilization for both the volatile oil droplets and the nanoprobes, thereby suppressing both microscopic coalescence and macroscopic phase breakdown during extended storage.

The comparative DFT analysis of the REO-emulgel, CD-emulgel, and smart-emulgel provides valuable insights into the electronic stability and synergistic interactions within these complex matrices (Figure 2e, Table 2). The frontier molecular orbital data revealed that the CD-emulgel possesses the widest energy gap (E_g_ = 0.5390 eV), followed by the smart-emulgel (E_g_ = 0.3537 eV), and the REO-emulgel (E_g_ = 0.3121 eV). This trend suggests that the incorporation of nitrogen-doped carbon dots enhances the kinetic stability of the system, whereas the lower E_g_ values observed for the REO-containing formulations indicates a more reactive electronic environment that may facilitate the interactions required for antimicrobial activity and pH-responsive sensing.

The thermodynamic stability of the systems was further evidenced by their total energy (E_T_) values: the smart-emulgel exhibited the lowest value (−3671.86 au), compared to −3087.72 au for the REO-emulgel and −2603.01 au for the CD-emulgel. This significant decrease in total energy for the smart-emulgel confirmed that the simultaneous integration of REO and QS-CDs into the chitosan-CMC matrix results in the most energetically favorable and stable composite configuration. Furthermore, the dipole moment (µ) values showed a clear correlation with the experimentally observed macroscopic stability. While the REO-emulgel exhibited a high dipole moment of 21.04 Debye, the smart-emulgel maintained a balanced polarity of 13.76 Debye, essential for the nanocomposite complex stabilization mechanism, ensuring the effective orientation of the amphiphilic polymers at the oil–water interface. The chemical reactivity parameters, including global hardness and electrophilicity, provide a theoretical basis for the smart functionality of the final material. The CD-emulgel exhibited the highest chemical hardness (0.2695 eV), suggesting a robust resistance to electronic perturbation. In the smart-emulgel, this value slightly decreased to 0.1768 eV, reflecting a system that remains stable during storage but electronically primed to respond to external stimuli.

To assess the thermodynamic stability of the emulgel assemblies, we calculated the binding energy (∆E_CD-emulgel_ for CD-emulgel and ∆E_smart-emulgel_ for smart-emulgel) of the complexed systems rather than raw total energies. The binding energy was determined using the following equation:

∆E_CD-emulgel_ = E_CD-emulgel_ − (E_polymer_ + E_QS-CDs_) (1)

∆E_smart-emulgel_ = E_smart-emulgel_ − (E_polymer_ + E_QS-CDs_ + E_REO_) (2)

where E_complex_ represents the total ground-state energy of the optimized assembly, and E_polymer_, E_QS-CDs_, and E_REO_ represent the optimized energies of the CMC/chitosan matrix, the nitrogen-doped QS-CDs, and the REO oil components, respectively. The DFT calculations yielded significantly negative ∆E_b_ for both systems (−3.33 au for CD-emulgel and −3.36 for smart emulgel), confirming that the integration of the components into the CMC/chitosan framework is a thermodynamically spontaneous and stable process. The marginal difference in binding energy between the two systems suggests that the inclusion of the REO core does not destabilize the assembly; rather, it participates in a stable, multi-component interfacial arrangement that is energetically favorable.

These DFT findings collectively validate the superior performance of the smart-emulgel, demonstrating that the calculated descriptors suggest favorable interactions among model fragments which are optimally balanced to provide a stable, yet highly sensitive, platform for real-time bacterial detection. Also, it is important to note that these DFT calculations are based on simplified molecular models and do not represent the full, complex, and hydrated emulgel system. Consequently, while these computational findings provide valuable insights into the fundamental molecular interactions, electronic compatibility, and chemical reactivity, they serve as a theoretical complement rather than an absolute proof of the macroscopic colloidal stability, microbial sensing kinetics, or long-term antimicrobial performance observed experimentally.

The biological efficacy of the formulated emulgels was assessed by monitoring the growth inhibition of *E. coli* and *S. aureus* using Optical Density (OD) measurements. As shown in the comparative analysis (Figure 2f), all formulations exhibited strong antibacterial activity, with inhibition rates consistently exceeding 70%. Previous studies demonstrated that REO owns remarkable anti-quorum sensing properties, mainly due to the presence of terpinyl acetate and 1,8-cineole, able to interfere with the pJBA132 sensor plasmid in *E. coli* models [51]. These results suggest that the developed emulgel does not merely act as a growth inhibition barrier but also disrupts the intercellular communication pathways required for pathogen coordination.

The REO-emulgel maintained high antibacterial efficacy against *E. coli* (79.66% inhibition), validating that the engineered low-substitution DS 0.4 CMC effectively anchors at the oil–water interface to preserve the bioactive volatile compounds of the REO oil. Rather than operating via a classical nanocomposite complex stabilization mechanism dependent on isolated rigid solid spheres, the amphiphilic polymer framework co-adsorbs at the boundary alongside the nanoprobes, establishing a structured viscoelastic nanocomposite barrier. Direct microstructural evidence of this assembly is revealed by comparing the TEM profiles. While the baseline REO-emulgel (Figure 2a) displays a standard clear phase boundary around the encapsulated droplet (605.49 nm), the smart-emulgel system (Figure 2c) exhibits a highly defined, electron-dense (darkened) perimeter surrounding the encapsulated oil droplet. This localized mass-thickness contrast suggests that the QS-CDs (3.31–6.75 nm) are tightly anchored within the zipped chitosan-CMC polyelectrolyte matrix directly at the oil–water interface. The formation of this dense, rigid nanocomposite shell effectively suppresses oil droplet coalescence at the contamination site. A primary observation from the data is the pronounced susceptibility of *E. coli* across all three formulations, with the CD-emulgel achieving the highest individual inhibition rate (83.67%). This suggests that the pomegranate-derived QS-CDs are not merely passive fluorescent probes: indeed, their ultra-small nanometric size (4.71–6.62 nm) and high density of nitrogen-doped surface groups allow them to directly interact with the lipopolysaccharide (LPS) layer of the Gram-negative bacteria cell wall, potentially inducing membrane destabilization and localized oxidative stress. In contrast, *S. aureus*, protected by its thicker, multi-layered peptidoglycan cell wall, exhibited higher resistance to the individual formulations. The resistance of *S. aureus* cannot be solely attributed to the thickness of its peptidoglycan layer. While Gram-positive bacteria lack an outer membrane, they remain notably susceptible to essential oils. Susceptibility is a multifaceted phenomenon governed by the physicochemical properties of the EOs and the physiological state of the bacteria. Key determinants include the lipid composition of the cytoplasmic membrane, the net surface charge of the cell, and the metabolic activity of the organism. Furthermore, the porous nature of the peptidoglycan layer allows for the diffusion of lipophilic EO components, which subsequently disrupt the cytoplasmic membrane’s integrity, alter its permeability, and impair vital metabolic functions, rather than being impeded by the peptidoglycan barrier alone.

Despite these challenges, our smart-emulgel demonstrated its synergistic potential as an integrated sensing-and-protection platform, achieving the highest inhibition rate against this Gram-positive strain (73.99%), compared to both the REO-emulgel (71.45%) and CD-emulgel (70.10%). This superior antibacterial performance of the smart emulgel is not merely additive but arises from a multi-pronged synergistic attack where each component plays a mechanical role in pathogen inhibition. The encapsulated REO core acts as a hydrophobic driver capable of targeting and permeabilizing the lipid-rich components of the bacterial membrane, while the embedded nitrogen-doped QS-CDs exert a complementary electronic disruption at the molecular interface. Both these active components are seamlessly immobilized within the polyelectrolyte matrix, and, in this nanocomposite architecture, the sugarcane bagasse-derived CMC ensures that the REO droplets remain dispersed without creaming over time, maintaining constant antimicrobial activity over time. Collectively, these biological data confirm that integrating nitrogen-doped carbon dots and essential oils within an upcycled sustainable polymer matrix generates a multifunctional platform that combines structural stability, antimicrobial protection, and sensing capability, offering a robust and green solution for real-time global food safety monitoring.

### 2.3. Optical Study

#### 2.3.1. pH-Dependent Photoluminescence with Visual pH-Responsive Behavior and Structural Stability

**Figure 3:**
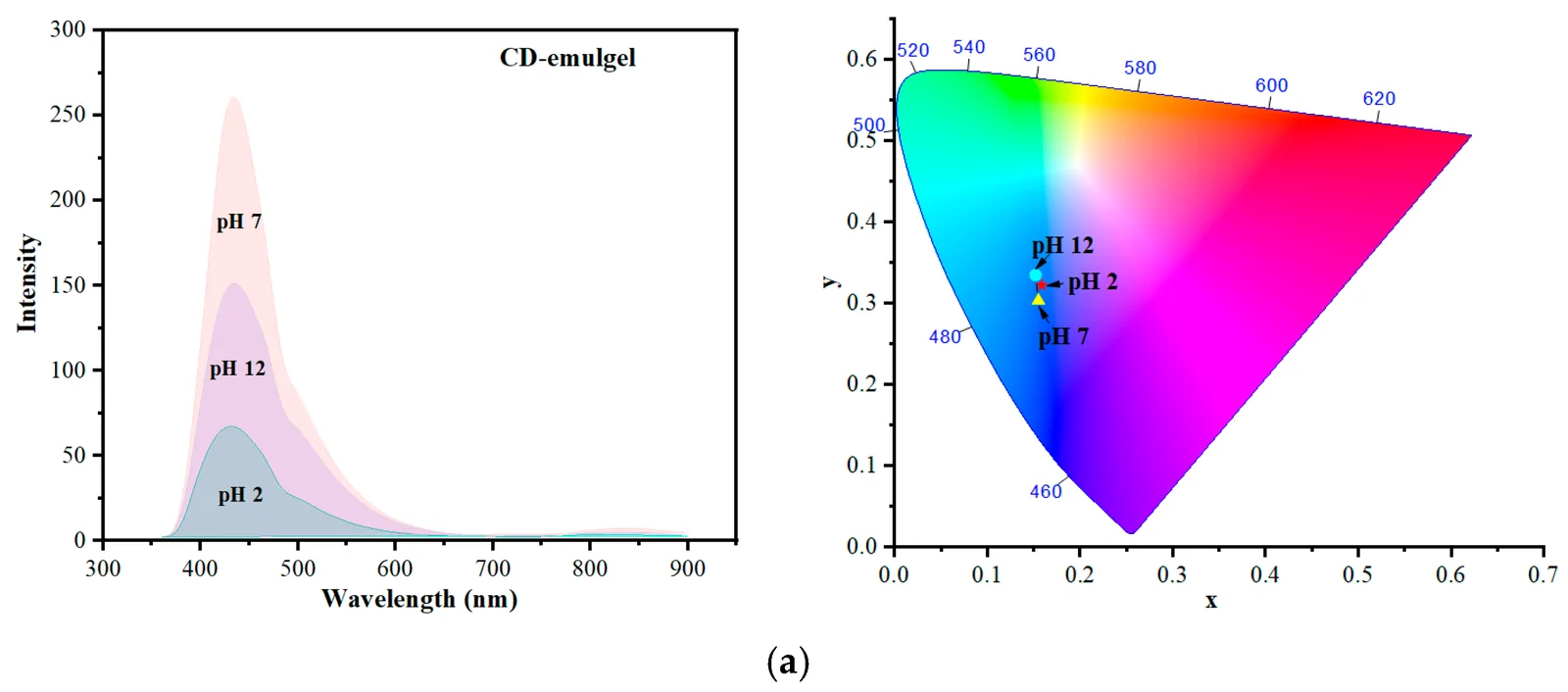

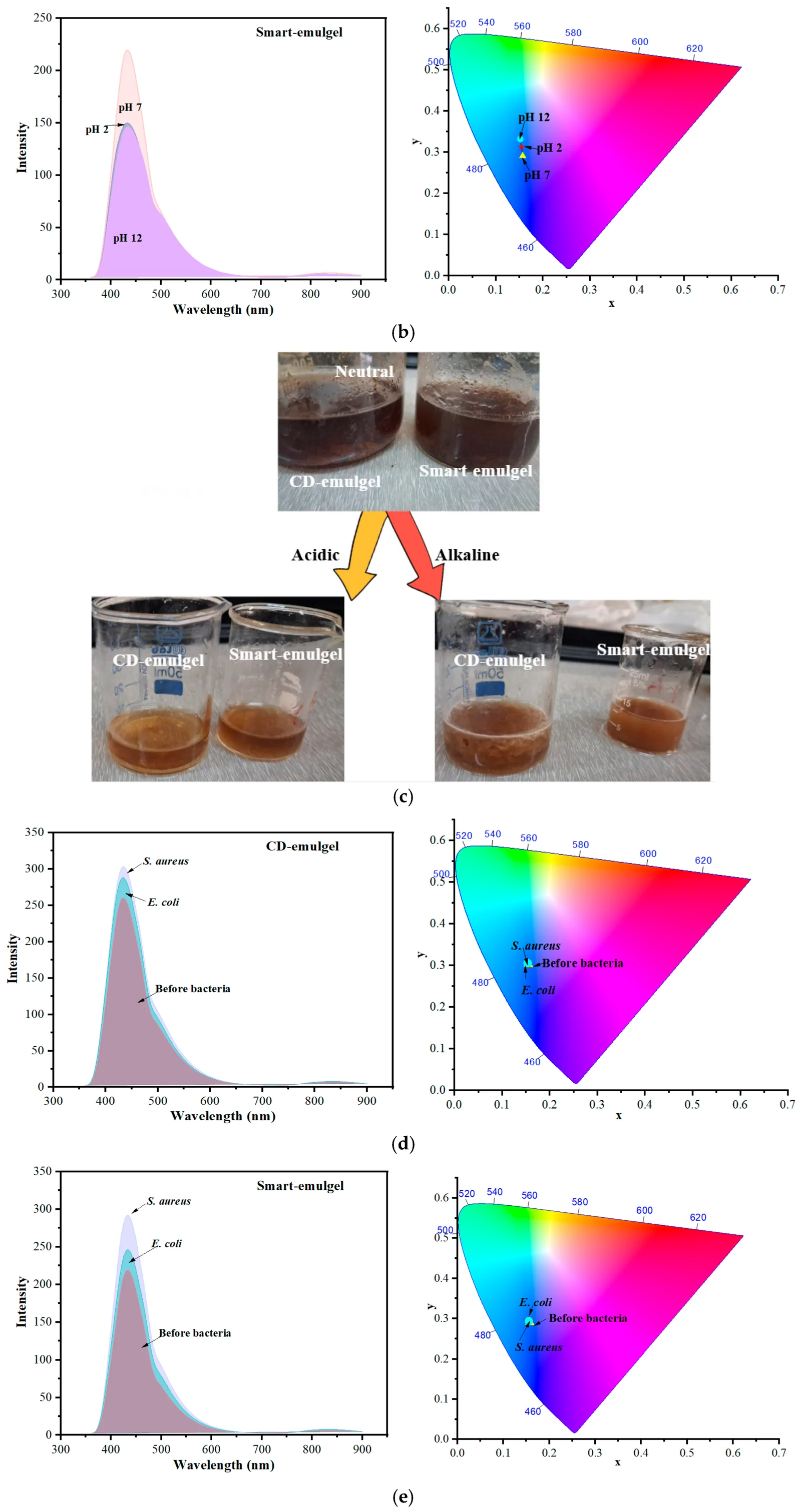
Fluorescent analysis for (**a**) CD-emulgel and (**b**) Smart-emulgel at different pH values, (**c**) Visual observation of the CD-emulgel and smart emulgel at different pH values, and fluorescent analysis for (**d**) CD-emulgel and (**e**) Smart-emulgel before and after contact with bacteria.

It is important to clarify that the smart-emulgel functions as a pH-responsive indicator of metabolic activity rather than a direct pathogen-specific detection system. Microbial proliferation typically results in the localized release of acidic or alkaline metabolites, a byproduct of bacterial respiration and enzymatic degradation of the food matrix. The smart-emulgel detects these shifts in the chemical environment as a proxy for the presence and proliferation of microorganisms. The colorimetric response of the smart-emulgel was evaluated across a broad pH range (2.0–10.0). Experimental data at pH 2 and pH 12 served as extreme calibration limits to define the full dynamic range and structural responsiveness of the nanocomposite matrix under high-stress conditions. Although these values lie outside the typical range of food spoilage, they demonstrate the high chemical resilience and the broad-spectrum responsiveness of the nitrogen-doped QS-CDs. The distinct colorimetric transitions observed at these extremes provide a high signal-to-noise ratio, ensuring that the sensor can accurately detect the more gradual acidification (due to organic acid production) and alkalization (due to volatile amine) characteristic of microbial contamination in real-time food monitoring applications (Figure 3).

It must be explicitly emphasized that these extreme values were selected strictly as operational boundary controls to evaluate the maximum colorimetric and fluorometric switching limits, total chemical reversibility, and saturation states of the active functional groups. At pH 2, exhaustive protonation of the carboxylate domains along the low-substitution CMC backbone (1643 cm^−1^ → 1733 cm^−1^) and of the amino groups on the QS-CDs was achieved, while at pH 12 complete deprotonation occurred. Although these boundary conditions do not represent the real narrower physiological pH fluctuations typically associated with natural food spoilage (pH 4.5–7.0), they provide the essential baseline calibration points required to interpret subtle intermediate transitions. The practical efficacy of the system under realistic food spoilage conditions (pH 4.5–7.0) is directly validated by the real-time in vitro experiments with *E. coli* and *S. aureus*. Crucially, this responsive behavior shows that modulating localized proton/deuteron transfer kinetics and suppressing volatile solvent activity can greatly stabilize functional interfaces and govern dynamic phase behaviors [52]. In the developed system, the highly responsive electronic structure of the smart-emulgel, as supported by the narrow calculated DFT energy gap (0.3537 eV), enabled efficient conversion of local ionic fluctuations into macroscopically readable optical signals. Accordingly, bacterial growth activity was directly translated into readable colorimetric and fluorescence modulations, confirming the suitability of the developed material as real-time packaging indicator for global food safety surveillance.

Both CD-emulgel and smart-emulgel exhibited a characteristic dual-emission profile, with primary peaks located in the blue and green regions of the visible spectrum, arising from the nitrogen doping of the carbon dot precursors. For the CD-emulgel, the emission peaks were recorded at 430/496 nm at pH 2, slightly shifting to 435/499 nm at pH 7, and stabilizing at 436/496 nm at pH 12. This subtle red-shift at neutral pH indicates the optimal stabilization of QS-CDs surface states within the chitosan–CMC polyelectrolyte matrix under maximum electrostatic interaction. The intensity trend for this system follows the order pH 7 > pH 12 > pH 2, where the maximum intensity at neutrality suggests that the dots are in their most “emissive” state. The significant quenching observed at pH 2 is likely due to the protonation of nitrogen-containing surface groups, which facilitates non-radiative recombination pathways and possible localized dots aggregation within the acidified polymer matrix. In the smart-emulgel, the REO incorporation modulates the optical response, shifting the emission peaks to 434/501 nm (pH 2), 431/494 nm (pH 7), and 433/499 nm (pH 12). Interestingly, the smart-emulgel exhibits a unique intensity order, namely, pH 7 > pH 2 > pH 12, indicating that, while neutral conditions remain the most favorable for fluorescence, the system retains significantly higher intensity under acidic conditions (pH 2) than alkaline conditions (pH 12). This deviation from the CD-emulgel’s behavior suggests that the REO droplets, stabilized by the low DS-CMC (DS = 0.4) matrix, create a protective hydrophobic microenvironment that partially shields the encapsulated QS-CDs from proton-induced quenching, preserving the sensors’ emissive properties under acidic stress. Conversely, the reduced intensity at pH 12 is attributed to the deprotonation of the chitosan–CMC framework, which disrupts the electronic coupling between the nitrogen-doped dots and the polymer matrix. These fluorometric results are fully aligned with the FTIR findings and supported by DFT data. The chemical handle identified by the carboxylate/carbonyl-related bands detected in FTIR at 1733 cm^−1^ represent the primary driver of these pH-triggered shifts. Under acidic conditions, the protonation of these groups supported by the narrow bandgap (E_g_ = 0.3537 eV) and low total energy (E_T_) found in the smart-emulgel’s DFT profile allows for sensitive electronic transduction. This confirms that the smart-emulgel is not merely a passive carrier but a responsive diagnostic tool. Its ability to maintain distinct optical signals at pH 2, combined with the antimicrobial shield provided by the REO, validates its potential as a dual-functional “sensing-shield” for real-time monitoring of food freshness and pathogen detection.

The stability and transition of the emulgels’ emission colors were further characterized using the CIE 1931 chromaticity diagram. For the CD-emulgel, the coordinates remained remarkably stable at (0.15, 0.32) for pH 2, (0.15, 0.30) for pH 7, and (0.15, 0.33) for pH 12, falling within the blue-cyan region of the CIE space. The constant x-coordinate (0.15) across the entire pH range indicates that the primary emission wavelength is structurally robust within the chitosan–CMC matrix. However, the slight fluctuation in the y-coordinate suggests a subtle modulation in color purity (saturation), where the neutral pH (0.30) represents the most focused emission, correlating with the highest fluorescence intensity previously observed. In the smart-emulgel, the CIE coordinates were recorded as (0.15, 0.31) at pH 2, (0.15, 0.29) at pH 7, and (0.15, 0.33) at pH 12. Similar to the CD-emulgel, the x-coordinate remains fixed at 0.15, confirming that the nitrogen-doped surface states of the N-doped QS-CDs dictate the fundamental blue emission.

Notably, the lowest y-coordinate at pH 7 (0.29) corresponds to the most saturated blue emission under optimal electrostatic stabilization. The increased y-coordinate (0.33) at pH 12, reflects a shift toward the green-cyan region, with macroscopic manifestation of the electronic deprotonation occurring at the interface of the smart matrix, as predicted by the DFT electrophilicity and bandgap analysis. The high stability of the (x, y) coordinates, particularly the constant x-value, is a significant advantage for real-time monitoring applications. It demonstrates that while the intensity of the signal significantly changes to indicate bacterial presence, the identity of the signal remains a clear, identifiable blue-cyan light, preventing false positives that could arise from color distortion. The calculated CIE coordinates derived from the fluorescence emission spectra provide a precise mathematical mapping of the emulgel’s optical response during active microbial monitoring (Figure 3). While the absolute numerical variations between the coordinates for the *Escherichia coli* and *Staphylococcus aureus* treatments occupy a narrow zone within the color space, these differences represent instrument-resolved, reproducible trends rather than arbitrary experimental noise. The high-resolution optical tracking system operates with an inherent sensitivity limit far tighter than the documented decimal variances, validating the physical authenticity of the shifts.

Chemically, these distinct localized positions in the CIE diagram are governed by the fundamentally different metabolic kinetics and organic acid secretion pathways characteristic of Gram-negative and Gram-positive pathways. The mixed-acid fermentation pathway of *E. coli* yields a faster, more aggressive accumulation of localized hydronium ions (H_3_O^+^) compared to the slower acidification rate of *S. aureus*. This kinetic divergence results in a distinct degree of protonation across the amine (–NH_2_) and carbonyl surface states of the embedded QS-CDs and the low-substitution CMC framework, as supported by our frontier molecular orbital predictions (energy gap of 0.3537 eV). Consequently, although these trends are presented as fundamental kinetic profiles rather than macro-scale statistical distributions, they successfully demonstrate that the smart-emulgel possesses the r electronic sensitivity to track and distinguish the real-time metabolic activities of diverse bacterial contaminants.

The transition in the macroscopic appearance of the smart-emulgel provides a robust, instrument-free mechanism for monitoring metabolic signatures (Figure 3b). As illustrated in the visual samples, the emulgel undergoes a dramatic color shift as the environment moves from acidic to alkaline. In the acidic phase (pH 2.0), the emulgel appears pale, translucent peach-yellow, due to the protonation of the carboxylate groups on the CMC and the amine groups on the chitosan. The resulting electrostatic repulsion leads to a less dense distribution of the QS-CDs and REO droplets, allowing more light to pass through the matrix. The transition in the macroscopic appearance of the smart-emulgel provides a robust, instrument-free mechanism for monitoring metabolic signatures. As illustrated in the visual samples, the emulgel undergoes a dramatic color shift as the environment moves from acidic to alkaline. In the acidic phase (pH 2.0), the emulgel appears pale, translucent peach-yellow, due to the protonation of the carboxylate groups on the CMC and the amine groups on the chitosan. The resulting electrostatic repulsion leads to a less dense distribution of the QS-CDs and REO droplets, allowing more light to pass through the matrix. This lightness in color signifies the initial stages of metabolic acidification typically associated with early-stage pathogen proliferation. Upon reaching the neutral phase (pH 7.0), the emulgel becomes deep amber-brown, corresponding to maximum electrostatic complexation (electrostatic zipping) between chitosan and CMC and the optimal localization of QS-CDs at the REO/water interface. This concentration of nitrogen-doped nanostructures and essential oil droplets increases light absorption and scattering, resulting in the dark, saturated appearance which correlates with the highest fluorescence intensity recorded in the fluorometric analysis. In highly alkaline conditions (pH 12.0), the emulgel shifts to a translucent reddish-brown or terracotta color, due to the deprotonation of the chitosan polymer chains and the consequent loss of the positive charge on the chitosan, leading to partial network relaxation. This structural loosening facilitates a change in the refractive index, increasing light scattering and shifting appearance toward a lighter reddish-brown tone, and a slight redistribution of the QS-CDs, leading to the observed shift in both visible color and the previously noted y-coordinate increase in the CIE space.

The visual evidence provided by the physical samples further validates the high degree of pH-responsiveness inherent in both the CD-emulgel and smart-emulgel systems, where the transition from neutral to extreme pH environments triggers significant chromotropic shifts and structural alterations (Figure 3c). Under neutral conditions, both emulgels exhibit a deep, translucent amber-to-dark brown appearance, indicating a stable and homogenous dispersion of the nitrogen-doped QS-CDs within the polyelectrolyte matrix. However, the introduction of acidic and alkaline stimuli reveals a divergence in their colloidal stability and optical signatures. Upon acidification, both systems undergo a noticeable lightening in color, transitioning toward a paler, yellowish-amber color; this shift is consistent with the protonation of the carboxylate groups on the CMC and the amino groups on the carbon dots, which effectively alters the electronic transitions of the QS-CDs to produce a macroscopic indicator of environmental acidification. The response under alkaline conditions highlights a critical structural distinction between the two formulations, as the CD-emulgel develops a distinctively turbid and highly viscous “shaded” texture compared to the smart-emulgel. This increased turbidity in the oil-free CD-emulgel suggests a localized aggregation or a salting-out effect of the polyelectrolyte complex as the chitosan chains deprotonate and lose their solubility in the basic medium. In contrast, the smart-emulgel appears significantly more resistant to this macroscopic phase transition, where the presence of the REO stabilized by the nanocomposite complex stabilization g-like mechanism of the DS 0.4 CMC likely acts as a structural buffer. These oil droplets effectively “interlock” within the polymer network, preventing the severe aggregation and excessive viscosity seen in the binary system. This macroscopic observation reinforces the findings from the DFT and TEM analyses, confirming that the smart-emulgel can maintain its structural integrity while providing a clear optical signal, making it a superior, robust platform for detecting the alkaline metabolic footprints often associated with protein degradation and fish spoilage.

#### 2.3.2. Real-Time Monitoring of Bacterial Metabolic Signatures

To evaluate the smart optical responsive capabilities of the developed matrix, the dual-mode (colorimetric and fluorometric) signaling efficiency was monitored during the active proliferation of *E. coli* and *S. aureus*. It should be explicitly clarified that the underlying mechanism of this optical transition is not driven by species-specific biological affinity or taxonomic identification. Instead, the composite acts as a highly sensitive interface that responds to the generalized chemical environment modifications induced by bacterial growth. As these model food pathogens are involved in active nutrient metabolism under localized packaging conditions, they secrete a continuous influx of short-chain organic acid byproducts (e.g., lactic, acetic, and succinic acids).

This localized metabolic accumulation drives down the local pH, triggering the immediate protonation of the un-ionized carbonyl groups (1733 cm^−1^) and carboxylate domains (1643 cm^−1^) of the low-substitution CMC backbone, as well as the nitrogen-rich surface groups (–NH_2_) of the embedded QS-CDs. The resulting alterations in surface charge distribution modulate the radiative recombination pathways of the carbon dots, converting microbial growth into measurable colorimetric and fluorometric responses. Although this transduction mechanism represents a generalized response to microbial acidification rather than selective pathogen isolation, such broad-spectrum responsiveness is highly advantageous for smart food-packaging applications. It ensures that the material acts as an early-warning indicator capable of signaling the proliferation of diverse acidogenic spoilage organisms and pathogens long before macroscopic decay becomes noticeable.

To properly contextualize the optical response of the smart-emulgel, the system’s baseline state prior to microbial proliferation must be considered as an internal sterile control. During the initial hours of incubation (represented by the Day 0 baseline), the emulgel was exposed to the uninoculated nutrient medium. The absolute stability of both the colorimetric profiles and the CIE coordinates under these conditions confirms that the chemical constituents of the sterile medium do not interfere with the sensing mechanism or induce false-positive optical responses. Furthermore, the smart-emulgel was intentionally engineered as a broad-spectrum metabolic responder rather than a taxonomically selective sensor. The matrix contains no species-specific biological receptors; therefore, its optical switching behavior is driven exclusively by the metabolic byproducts generated during microbial growth, including volatile amines and short-chain organic acids. Although the absence of species-level selectivity limits the material’s application as a diagnostic identification tool, this broad responsiveness is a critical asset for commercial smart-packaging applications. Real-world food spoilage is inherently unpredictable and rarely limited to a single bacterial strain. By responding to generalized metabolic acidification and alkalization gradients, the smart-emulgel translates microbial proliferation into a readily detectable optical alert, thereby improving food-quality monitoring and consumer safety.

The diagnostic transition of these materials from a steady state to an active alert mode highlights the interaction between the nanostructured matrix and microbial metabolism. In the CD-emulgel (the binary system), the baseline dual-emission at 435/499 nm shifted to 434/496 nm in the presence of *E. coli* and to 434/498 nm in the presence of *S. aureus*. This rapid electronic response is characteristic of the nitrogen-doped QS-CDs’ sensitivity to the subtle acidification of their microenvironment (Figure 3d). In the smart-emulgel, the baseline emission was observed at 431/494 nm, reflecting the influence of the encapsulated REO on the matrix’s refractive index. Upon contact with *E. coli*, the emission showed a minimal shift to 432/494 nm, suggesting that the protective zipped polyelectrolyte network effectively buffers the carbon dots from immediate metabolic interference. However, the response to *S. aureus* was markedly different, triggering a significant bathochromic (red) shift to 435/500 nm (Figure 3e). This distinct red-shift in the smart-emulgel contrasting with the blue-shift in the CD-emulgel indicates that the presence of the REO droplets alters the transduction pathway. It appears that the metabolic byproducts of *S. aureus* may induce a localized swelling or relaxation of the CMC/chitosan–oil interface, allowing the carbon dots to probe a different electronic environment.

Crucially, both formulations exhibited a consistent “OFF-to-ON” fluorescence enhancement upon bacterial growth, with emission intensity following the order:

*S. aureus* > *E. coli* > Sterile Baseline

The fact that *S. aureus* consistently triggered the highest intensity across both formulations suggests that the Gram-positive metabolic signature, likely a higher concentration of lactic acid and specific extracellular enzymes, interacts more synergistically with the pomegranate-derived nitrogen groups. In the smart-emulgel, the fluorescence enhancement ensures that, even as in the presence of the REO antimicrobial phase, the material is able to maintain its sensing functionality, providing a bright, unmistakable optical signal. The smart-emulgel enables the discrimination of specific pathogens through discrete bathochromic shifts in its fluorescence spectra. Unlike conventional passive packaging, this stimuli-responsive matrix functions as an integrated sensing platform, providing autonomous, real-time monitoring of food safety through quantifiable optical signal transduction.

### 2.4. Thermal Analysis with DSC

**Figure 4:**
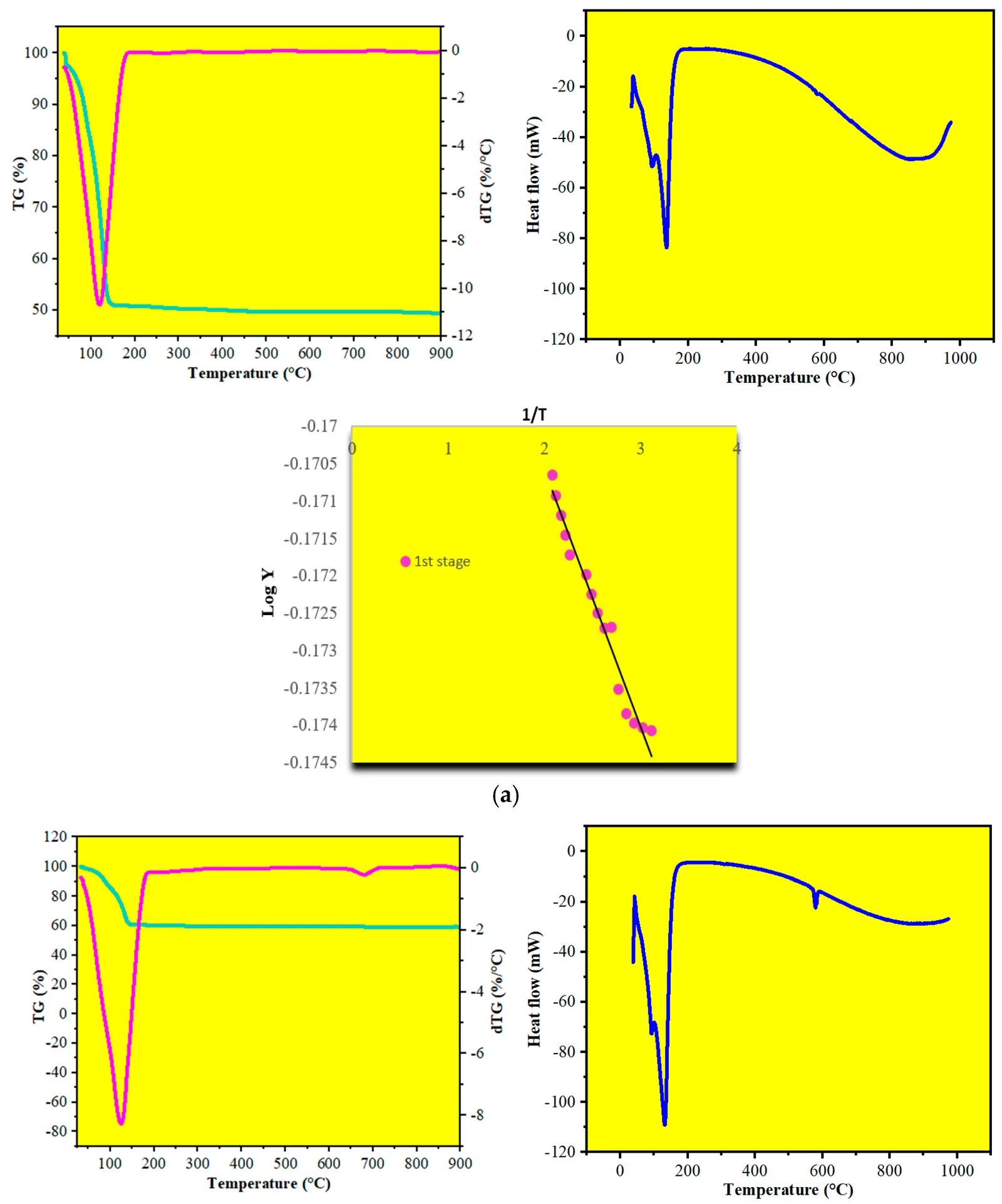

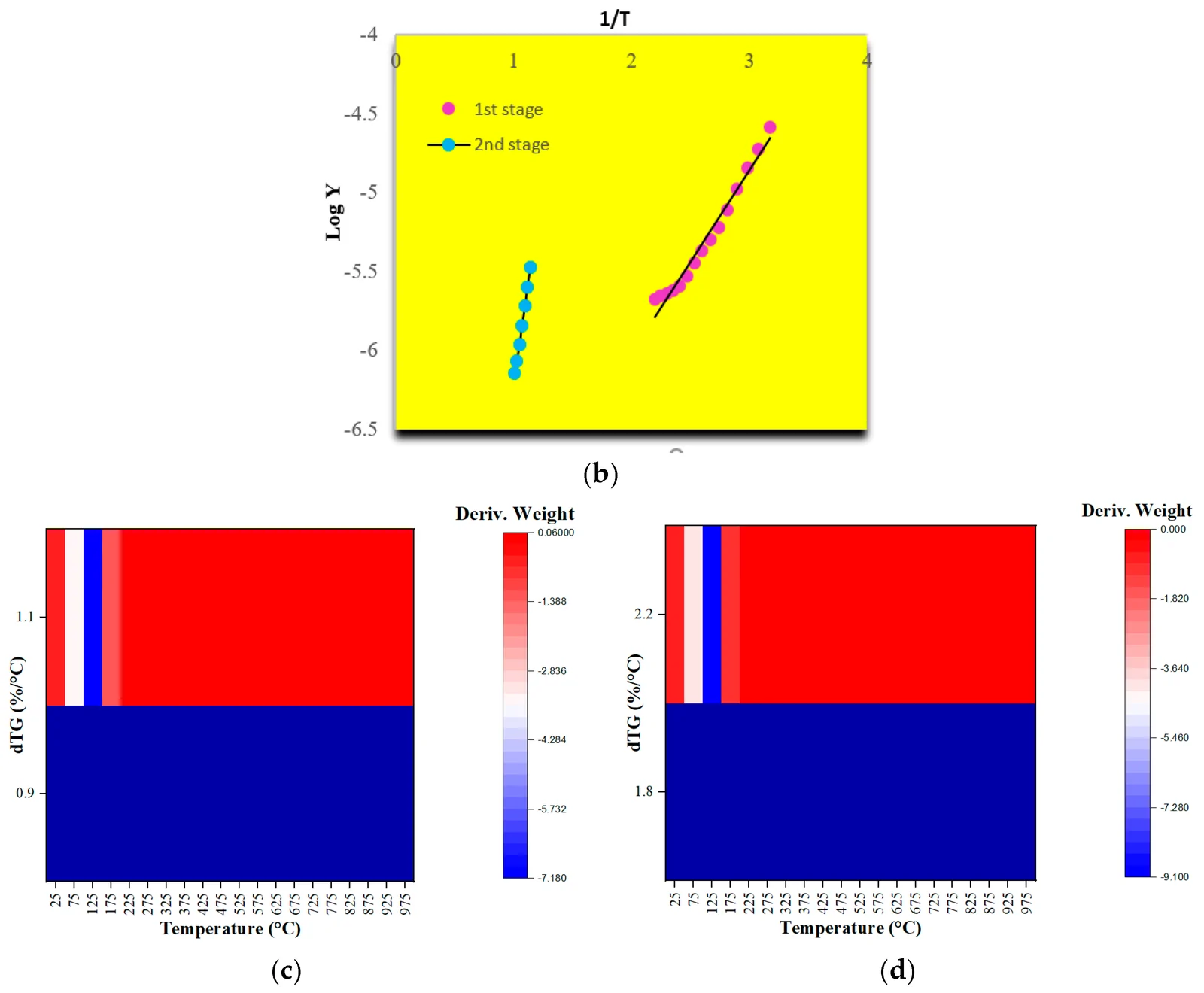
TGA/DSC for (**a**) CD-emulgel and (**b**) Smart-emulgel and heat map for (**c**) CD-emulgel, and (**d**) Smart-emulgel.

The thermogravimetric analysis (TGA) and derivative thermogravimetry (DTG) data provide key insights into the structural transformations occurring upon REO incorporation into the carbon dot-polyelectrolyte matrix (Figure 4, Table 3). For the CD-emulgel, a clear two-stage decomposition profile was observed. The first stage, occurring between 48.23–208.21 °C with a DTG peak at 118.75 °C, involved a significant mass loss of 23.09%. This is primarily attributed to the evaporation of physically adsorbed and chemically bound water molecules trapped within the chitosan–carboxymethyl cellulose (CMC) matrix, alongside the initial dehydration of the oxygenated surface groups of the QS-CDs.

On the contrary, the smart-emulgel showed a two-stage degradation profile, with DTG peaks observed at 125.43 °C and 679.60 °C. The first stage is attributed to the evaporation of physically adsorbed and chemically bound water, while the second stage, occurring at the significantly higher temperature of 679.60 °C, represents the final carbonization of the polymer backbone and the degradation of the thermally stable, integrated network. The presence of these two distinct stages indicates a segmented matrix where water-binding sites and the carbonized skeleton are thermally decoupled, requiring individual activation energies (E*_a_*) of 22.12 kJ/mol and 95.19 kJ/mol, respectively. This marked increase in the activation energy for the second stage, compared to the corresponding stage in the CD-emulgel, suggests a substantial enhancement in thermal stability following REO incorporation. Indeed, the incorporation of REO does not simply dilute the matrix but also promotes the formation of a more cohesive and structurally integrated network, where the amphiphilic CMC (DS = 0.4) and cationic chitosan establish a hyper-stable shell capable of effectively immobilizing both the REO and QS-CDs into a single, unified thermodynamic entity. The shift in the reaction order to *n* = 1.5 for the smart-emulgel suggests a more complex degradation mechanism compared to the first-order kinetics (*n* = 1) observed in the CD-emulgel. This fractional reaction order indicates that the degradation process is no longer purely diffusion-controlled but is instead governed by the structural constraints of the integrated nanocomposite network. The increased complexity of this kinetic model supports the hypothesis that the assembly behaves as a unified thermodynamic entity, where the degradation of the polyelectrolyte backbone is hindered by the densely packed, cross-linked architecture.

The thermodynamic parameters calculated via the Coats–Redfern model provide a quantitative basis for the observed structural stability. Specifically, the increase in activation energy (E_a_) and the associated pre-exponential factors indicate a more rigid molecular framework, consistent with the intermolecular cross-linking between the CMC and chitosan chains. The Gibbs free energy (∆G) for the smart-emulgel is remarkably high (⅀G = 428.89 kJ/mol), confirming that the degradation process is highly non-spontaneous and the system is in an extremely stable state. The massive positive enthalpy (∆H = 106.1 kJ/mol) reflects the enormous energy required to rupture the complex network of electrostatic and hydrogen bonds holding the oil droplets and nitrogen-doped carbon dots within the polymer mesh. Furthermore, the negative entropy values (∆s = −0.23 and −0.24) for both formulations indicate that the transition states are more ordered than the initial reactants, a characteristic feature of highly zipped polyelectrolyte complexes. Collectively, these data prove that the smart-emulgel is not merely a mixture but a high-performance nanostructured composite with exceptional resistance to thermal perturbation.

The DSC analysis complements the thermokinetic findings by elucidating the phase transitions and structural organization of the emulgel matrices (Table 4). The DSC thermograms of the smart-emulgel (Figure 4b) revealed high-temperature stability beyond the typical degradation range of organic polymers. While the initial endothermic peaks corresponded to moisture loss and polymer chain relaxation, the prominent exothermic event at 570 °C was identified as the maximum heat release associated with the oxidative degradation of the polyelectrolyte-nanocarbon network (T_exo,max_), associated with the final oxidative degradation of the zipped polyelectrolyte–nanocarbon network. The shift of this peak to 570 °C, compared to the lower decomposition thresholds of the precursors, highlights the enhanced thermal resistance of the system, which is a direct consequence of the electrostatic interactions between the cationic chitosan and anionic CMC. Both formulations exhibited two melting peaks (T_m1_ and T_m2_), reflecting the complex thermal response of the hydrated polyelectrolyte network. For the CD-emulgel, T_m2_ was observed at 137.06 °C, which slightly shifted to 132.13 °C after the incorporation of rosemary essential oil in the smart-emulgel. This modest decrease in the melting point is characteristic of the plasticizing effect of the lipid phase, which facilitates localized chain mobility. Notably, the shift in T*_exo,max_* from 572.11 °C to 579.48 °C upon integration of the REO and QS-CDs demonstrates the enhanced thermal stability of the matrix, which provides complementary evidence of the increased energy required for the degradation of this structurally constrained network, cross-validating the thermal robustness observed in our TGA/DTG analysis. The degree of crystallization (X*_c_*) calculated from the DSC thermograms reveals that the emulgel matrices are largely amorphous in nature. The X*_c_* value rose from 0.11 in the CD-emulgel to 0.14 in the smart-emulgel; while this indicates that the system remains predominantly amorphous, the increase suggests a slight enhancement in the formation of ordered molecular domains. This implies that the REO droplets, in coordination with the amphiphilic CMC (DS = 0.4), may act as localized nucleating sites, promoting a more organized arrangement of the chitosan-CMC chains compared to the control. Although the system does not form extensive crystalline lattices, these localized, more ordered domains contribute to the structural reinforcement of the matrix. This subtle increase in molecular order aligns with the enhanced kinetic stability observed in TGA, as these reinforced regions require slightly higher energy for thermal disruption compared to the more disordered segments of the CD-only matrix. A critical distinction observed in the DTG heat maps is the presence of a prominent white transition band at 75 °C in the CD-emulgel, which is significantly attenuated in the smart-emulgel formulation (Figure 4c,d). This white zone signifies a mid-range weight loss rate associated with the removal of bound moisture from the porous polyelectrolyte matrix. The suppression of this stage in the smart-emulgel suggests that the integration of REO occupies these interstitial spaces, effectively waterproofing the internal structure. Rather than the multiple mass-loss stages seen in the baseline, the smart-emulgel exhibits a refined degradation profile where the initial moisture loss is consolidated. This is followed by a high-intensity thermal degradation event centered at approximately 135 °C, which we attribute to the electrostatic zipping of the polyelectrolyte matrix. This, coupled with the final carbonization stage at 679.60 °C, confirms the formation of a thermally robust, segmented architecture. By utilizing CMC with a precisely engineered degree of substitution (DS 0.4), we’ve created an amphiphilic bridge. This bridge does not just hold the oil; it pulls the chitosan and CDs into a dense, semi-crystalline shell around each oil droplet. The substantial increase in activation energy reflects the greater resistance of this integrated architecture to thermal disruption.

### 2.5. Emulsion Stability and Polyelectrolyte Synergy

#### 2.5.1. The Role of HLB in Nanocomposite Complex Stabilization

The calculated Hydrophilic–Lipophilic Balance (HLB) values serve as a quantitative indicator of the relative changes in surface activity upon nanocomposite assembly. While absolute HLB values for polysaccharides are traditionally constrained by their inherent hydrophilicity, the increase in the calculated HLB from 1.40 in the REO-emulgel to 5.34 in the smart-emulgel reflects a strategic transition in surface activity. Rather than acting as a traditional small-molecule surfactant, the low-DS (0.4) CMC functions as an amphiphilic anchor: the lower degree of substitution increases its relative lipophilicity compared to high-DS counterparts, allowing it to preferentially localize at the oil–water boundary alongside cationic chitosan. This assembly, evidenced by our TEM observations, creates a stable barrier through steric and electrostatic stabilization rather than classical surfactant-based emulsification. Thus, the tuned HLB values provide a comparative metric demonstrating that our formulation moves from a system optimized for oil-phase anchoring toward a more balanced, multi-component stability. Upon incorporation of the nitrogen-doped QS-CDs, the HLB value of the smart-emulgel is tuned to 5.34, a value which falls within the optimal range (4–6) for stable multicomponent emulsions. This intermediate HLB indicates that, while the system maintains strong lipophilic anchoring to the oil core, it has gained sufficient hydrophilic character to remain compatible with the aqueous chitosan matrigel. The determined HLB confirmed the transition of the low-DS CMC (DS = 0.4) from a purely water-soluble polymer to a surface-active material. This specific amphiphilicity allows the CMC to provide hydrophobic anchoring points within the rosemary oil droplets, while the cationic chitosan electrostatically stabilizes the interface. The resulting synergistic interaction prevents droplet coalescence and provides a stable, structured environment for the pH-responsive QS-CDs, thereby ensuring the thermodynamic stability of the smart-emulgel without the need for synthetic surfactants. Thus, this balanced amphiphilicity contributes both to physical stability, preventing oil leakage, and to functional accessibility for external stimuli sensing.

#### 2.5.2. Polyelectrolyte Complexation and Structural Assembly

The structural stability of the system is driven by polyelectrolyte complexation between the cationic chitosan and anionic CMC. At the oil droplet interface, the protonated amine groups (–NH_3_^+^) of chitosan electrostatically interact with the carboxylate groups (–COO^−^) of the CMC, resulting in the formation of a dense, viscoelastic polyelectrolyte complex (PEC) shell. This process, which we describe as an ‘electrostatic zipping’ mechanism, effectively traps the antimicrobial REO core within a robust, three-dimensional network. The inclusion of QS-CDs does not disrupt this shell; instead, as supported by the DFT analysis, the nitrogen-rich surface groups of the dots show strong electronic compatibility with the CMC/chitosan matrix. The dots are effectively trapped within the zipped network, ensuring that they remain positioned at the droplet periphery. The optical sensing mechanism of this architecture is governed by the pH-dependent protonation/deprotonation of these nitrogen-rich surface functional groups. As microbial activity, such as *E. coli* and *S. aureus*, proliferate, they secrete organic acids into the food matrix, lowering the local pH. When the local pH drops below the characteristic pKa of the QS-CDs’ surface amine and amide moieties, a spontaneous protonation event occurs. This modification of the surface electronic state induces a rapid shift in the radiative recombination pathways of the carbon dots, triggering the observed colorimetric and fluorometric signals. Thus, the emulgel acts as a sensitive transducer of local hydronium ion concentration, providing a real-time monitor of microbial metabolic activity. This spatial arrangement is critical for functionality: the carbon dots are placed at the primary interface, where they can immediately sense, under the tested conditions, the metabolic pH variations associated with pathogens’ activity, while remaining structurally coupled to the antimicrobial REO core. The stabilization mechanism of the smart-emulgel is fundamentally tied to the tailored DS of the CMC backbone. The CMC with a high DS, conferring strong hydrophilicity and complete solubility in the aqueous phase, which generally limits its ability to stabilize oil-water interfaces without additional surfactants [34]. In contrast, the low DS (0.4) engineered in this study was intentional; it effectively optimizes the polymer’s Hydrophilic–Lipophilic Balance (HLB). By reducing the density of carboxylate groups, we decreased the polymer’s affinity for the bulk aqueous phase. This allows the amphiphilic CMC to act as a Pickering stabilizer, promoting the formation of a robust, zipped nanocomposite interface between the hydrophobic essential oil droplets and the aqueous matrix, ensuring structural stability without the need for synthetic surfactants.

#### 2.5.3. Thermal and Kinetic Stability (Coats-Redfern Insights)

The kinetic analysis, performed using the Coats–Redfern model, yielded distinct apparent activation energies (E*_a_*) for each degradation stage of the smart-emulgel. The first stage, centered at 125.43 °C, exhibited an E*_a_* of 22.12 kJ/mol, corresponding to the dehydration and moisture loss from the polymer matrix. The second stage, occurring at 679.60 °C, required a significantly higher activation energy of 95.19 kJ/mol, reflecting the substantial energy barrier for the carbonization and thermal breakdown of the integrated nanocomposite architecture. This pronounced increase in E_a_ for the second stage compared to the first confirms that the REO and QS-CDs have created a thermally robust, highly cross-linked network that requires significant thermal energy for complete structural disruption.

The extraordinary significant increase in activation energy (E_a_) observed for the smart-emulgel compared to the CD-emulgel (18.69 kJ/mol) can be directly attributed to the synergistic interactions among the three components, i.e., REO, QS-CDs, and the CMC/chitosan matrix. The addition of REO (lowering the HLB to 5.34) promoted a more compact and ordered packing of the polymer chains around the oil droplets. The Coats–Redfern analysis confirms that the smart-emulgel undergoes a multi-stage degradation process, reflecting the structural hierarchy of the nanocomposite. Rather than an independent loss of components, the two-stage profile (125.43 °C and 679.60 °C) indicates that the REO and QS-CDs are deeply integrated within the polymer framework, forming a structurally coupled system where the thermal degradation of the backbone is intrinsically linked to the interfacial architecture. These results suggest that the three components have transitioned from a simple mixture into a structurally integrated nanocomposite (X*_c_* = 0.14). This thermodynamic robustness, supported by the calculated Gibbs free energy (∆G) values, confirms a favorable, stable configuration. This ensures that the emulgel maintains its structural integrity and sensing accuracy despite the fluctuations in temperature and humidity typically encountered during food storage and transport. This thermodynamic robustness, validated by the negative activation energy (E_a_) values in the DFT calculations, confirms a favorable energetic configuration and ensures that the emulgel maintains its structural integrity and sensing accuracy under the temperature and humidity variations which typically occur during food storage and transport.

## 3. Conclusions

The present study successfully engineered a sustainable multifunctional smart-emulgel by integrating Pomegranate-derived nitrogen-doped carbon dots (QS-CDs) and Rosemary essential oil (REO) into a chitosan/carboxymethyl cellulose (CMC) polyelectrolyte matrix. The obtainment of low-DS CMC (0.4) from sugarcane bagasse was pivotal, as its unique amphiphilic character enabled effective stabilization of the REO droplets through nanocomposite complex formation, resulting in a unified and thermodynamically stable delivery and sensing platform. Transmission Electron Microscopy confirmed the ultra-small, quasi-spherical morphology of the QS-CDs (4.71–6.62 nm) and the formation of a dense, zipped shell surrounding the oil droplets in the smart-emulgel.

The smart-emulgel demonstrated a synergistic stabilization effect within the polymer matrix. While the CD-emulgel baseline exhibited a primary degradation stage with an activation energy (E*_a_*) of 18.69 kJ/mol, the smart-emulgel displayed a more complex two-stage degradation profile. Notably, the high-temperature second stage of the smart-emulgel required a significantly higher activation energy of 95.19 kJ/mol. This substantial increase in the energy barrier for the breakdown of the integrated backbone highlights the formation of a thermally robust, highly cross-linked network driven by the incorporation of REO. This result, coupled with an increase in the degree of crystallization (X_c_) from 0.11 to 0.14, underscores the formation of a robust system with restricted chain mobility and enhanced structural integrity.

While the diffuse halos detected in the SAED patterns primarily reflect the largely amorphous nature of the carbohydrate-based matrix, the observed structural evolution provides meaningful insights into the system’s integration.

The intensification of the diffraction rings in the smart-emulgel, compared to the REO-emulgel, suggests a localized increase in structural order, induced by electrostatic interactions between nitrogen-doped QS-CDs and CMC chains (DS 0.4), with the formation of coordinated clusters. This semi-amorphous structure is functionally vital, as it preserves the matrix flexibility required for real-time chromatic response while ensuring the thermal stability confirmed by the E_a_ values. Theoretical DFT calculations further validated these experimental findings, showing that the smart-emulgel possesses the most energetically favorable configuration (−3671.86 au) and an optimized electronic profile suitable for sensitive molecular transduction. Functionally, the smart-emulgel exhibits a dual-mode optical response, capable of detecting the metabolic pH signatures of *E. coli* and *S. aureus*, enabled by synchronized colorimetric transitions and fluorescence quenching of the N-doped QS-CDs. In conclusion, this research provides a high-performance, bio-based platform that combines antimicrobial activity with sensitive, smart monitoring, offering a significant advancement in the design of real-time food freshness indicators and clinical diagnostic tools.

## 4. Materials and Methods

### 4.1. Materials

Pomegranate peel waste was obtained from local markets in Egypt and utilized as the precursor for carbon dot synthesis. Sugarcane bagasse (SCB), used for the preparation of carboxymethyl cellulose (CMC), was generously supplied by the Quena Company (Qus City, Egypt) for Paper Industry, Egypt. Analytical grade urea and sodium hydroxide were procured from Sigma-Aldrich (St. Louis, MO, USA). All additional chemical reagents were of analytical grade and utilized without further purification.

### 4.2. Microwave-Assisted Synthesis of Quasi-Spherical Nitrogen-Doped Carbon Dots (QS-CDs)

Quasi-spherical carbon dots (QS-CDs) were synthesized from pomegranate waste via a rapid microwave-assisted hydrothermal route. Fresh pomegranate exocarp (skin) was finely chopped and homogenized. Subsequently, 4 g of the biomass was dispersed in 100 mL of deionized water, followed by the addition of 4 g of sodium hydroxide and 4 g of urea. In this reaction, sodium hydroxide served as a chemical activator to disrupt the lignocellulosic and anthocyanin matrices of the peel, while urea acted as the nitrogen precursor for surface doping. The mixture was subjected to microwave irradiation at 900 W for 10 min, facilitating rapid dehydration, polycondensation, and carbonization into ultra-small particles. After cooling to room temperature, the crude product was filtered to eliminate large carbonaceous aggregates.

### 4.3. Synthesis of Low-DS Carboxymethyl Cellulose

Following the protocol established by Tohamy et al. [19], cellulose was isolated from SCB. For the carboxymethylation process, 15 g of the extracted cellulose were dispersed in 400 mL of isopropanol. The mixture underwent alkalization for 60 min at room temperature by the dropwise addition of a 25% NaOH solution over a period of 30 min. Etherification was then initiated by the gradual addition of 18 g of monochloroacetic acid (MCA) in isopropanol over 30 min. The reaction was maintained at 55 °C for 3.5 h under continuous stirring. Upon completion, the liquid phase was decanted, and the resulting fibrous CMC was purified with 70% methanol, filtered, and then dried at 60 °C for 5 h until complete drying.

### 4.4. DS Characterization via Back-Titration

The Degree of Substitution (DS) was quantified through a back-titration method. First, 2 g of CMC was converted into its acid form (H-CMC) by dispersing it in a mixture of 37.5 mL of ethanol and 2.5 mL of HNO_3_, followed by boiling for 2 min and stirring for 15 min. The resulting H-CMC was washed six times with ethanol (80%) at 60 °C to remove impurities, and then dried at 105 °C for 3 h. For the titration assay, 1 g of the dried H-CMC was dissolved in 100 mL of deionized water and treated with 25 mL of 0.3 N NaOH. After boiling the mixture for 15 min, the unreacted NaOH was neutralized with 0.3 N HCl, using phenolphthalein as the endpoint indicator. The DS was then calculated using the molecular weight of the anhydroglucose unit of cellulose (162 g mol^−1^) and the net increase in molecular weight associated with each carboxymethyl substitution (58 g mol^−1^), according to the following equation:

(3) $$\mathrm{DS}\;=\;\frac{0.162A}{1-0.058\;A}$$

The coefficient A was determined according to the standard milliequivalent formula:

(4) $$A\;=\;\frac{BC\;-\;DE}{F}$$

where B and C represent the volume (ml) and normality of NaOH solution, respectively; D and E represent the volume (mL) and normality of HCl, respectively; F is the mass (g) of CMC used in the analysis; 162 = gm molecular weight of anhydroglucose unit of cellulose; 58 = net increase in molecular weight of anhydroglucose unit for each CMC substituted; and A corresponds to the milliequivalents of consumed acid per gram of sample [19].

### 4.5. Preparation of Amphiphilic Polyelectrolyte Emulgels

The emulgels were fabricated using a two-step process involving high-shear emulsification followed by electrostatic gelation. Three distinct formulations were prepared to evaluate the synergistic effects of the components:

- REO-emulgel: (1% CMC + 1.5% Chitosan + 1% Rosemary oil);
- CD-emulgel: (1% CMC + 1.5% Chitosan + 1% Quasi-spherical carbon dots (QS-CDs)); and
- Smart-emulgel: (1% CMC + 1.5% Chitosan + 1% Rosemary oil + 1% QS-CDs).

#### 4.5.1. REO-Emulgel

A 1% (*w*/*v*) CMC solution was prepared in deionized water. 1% (*v*/*v*) rosemary essential oil (REO) was added to the CMC solution. The mixture was homogenized at 12,000 rpm for 5 min to obtain an anionic pre-emulsion. Subsequently, a 1.5% (*w*/*v*) chitosan solution (in 1% acetic acid) was added dropwise under continuous stirring to induce gelation. The hydrophobic domains of the DS 0.4 CMC anchored to the REO droplets, while chitosan interconnected the droplets into a 3D network through electrostatic interactions. This formulation served as the baseline for antimicrobial activity evaluation.

#### 4.5.2. CD-Emulgel

1% (*v*/*v*) dispersion of the synthesized QS-CD was incorporated into a 1% (*w*/*v*) CMC solution. The mixture was stirred for 20 min to promote interactions between the nitrogen-rich groups of the CDs and the carboxylate groups of the CMC. Subsequently, a 1.5% (*w*/*v*) chitosan solution was added to form a translucent, fluorescent polyelectrolyte gel. The QS-CDs were physically entrapped within the polymer network. Owing to the absence of oil, this formulation exhibited maximum transparency and was used as the baseline for evaluating pH-responsive fluorescence and colorimetric shifts.

#### 4.5.3. Smart-Emulgel

The smart emulgel was prepared by first mixing a 1% (*w*/*v*) CMC solution with 1% (*v*/*v*) QS-CDs suspension. Rosemary essential oil (1% *v*/*v*) was then added, and the resulting mixture was homogenized at 15,000 rpm to obtain a QS-CD loaded anionic emulsion. Finally, a 1.5% (*w*/*v*) chitosan solution was added slowly until a thick and stable emulgel was formed. This formulation represents a multicomponent polyelectrolyte complex in which the REO droplets provide antimicrobial features, while the N-doped CQDs the smart sensing capability. The amphiphilic nature of CMC ensures the stable coexistence of the oil phase and the water-dispersible carbon dots, preventing phase separation.

### 4.6. Structural Characterization and HLB Calculation

#### 4.6.1. FTIR Spectroscopy and DS Quantification for CMC

The chemical functionality and coordination of the synthesized samples were analyzed by means of Fourier-transform infrared (FTIR) spectroscopy using a Mattson-5000 spectrometer (Unicam, Somerset, UK). Samples were prepared using the KBr pellet technique, and spectra were recorded over the wavenumber range of 4000 to 400 cm^−1^ at a high resolution. To complement the titrimetric method, the DS was also estimated from the relative intensity of the characteristic infrared absorption bands. The calculation was based on the ratio between the absorbance of the carbonyl group and that of the cellulose backbone, according to the following equation:

(5) $$\mathrm{DS}\;=\;\frac{A_{C=O}}{A_{C-O-C}}$$

where A_C=O_ represents the absorbance intensity of the carboxylate group and A_C–O–C_ the absorbance of the ether linkage within the glucose rings [53].

#### 4.6.2. Hydrophilic–Lipophilic Balance (HLB) for CD-Emulgel and Smart-Emulgel

The amphiphilic nature of the SCB-derived CMC, which governs its efficiency as an nanocomposite stabilizer, was quantified by determining its Hydrophilic–Lipophilic Balance (HLB) in combination with the chitosan matrix. This parameter is pivotal for predicting the affinity of the QS-CDs and the polyelectrolyte chains at the Rosemary essential oil/water interface. The HLB of the stabilizing system was determined according to the following equation [34]:

(6) $$\mathrm{HLB}\;=\;20\;\times \;\frac{M_{hydrophilic}}{M_{total}}$$

where M_hydrophilic_ corresponds to the combined molecular weight of the hydrophilic moieties, namely the CMC carboxylate groups (–COO^−^) and the chitosan protonated amine groups (–NH_3_^+^), while M_total_ represents the total molecular mass of the functionalized polymer chains.

#### 4.6.3. Evaluation of Metabolic Bacterial Detection and Pathogen Interaction

The biological sensing capability of the CD-emulgel and smart-emulgel was evaluated against two model pathogens representing primary threats in clinical health and food safety: the Gram-negative bacterium *Escherichia coli* and the Gram-positive bacterium *Staphylococcus aureus*. These microorganisms were selected to investigate how the functionalized N-doped surfaces of the emulgel matrices interact with the distinct cell wall architectures of different bacterial classes.

Bacteria were cultured in a suitable broth at 37 °C until reaching the exponential growth phase. To monitor metabolic pH variations, a standardized concentration of both CD-emulgel and smart-emulgel was introduced into the bacterial suspensions. The dual-mode optical response was assessed through the following two-stage experimental protocol.

#### 4.6.4. Direct Pathogen Contact Analysis (Biological Interface)

The effect of physical anchoring and surface interactions was investigated by introducing the emulgels into bacterial suspensions (*E. coli* and *S. aureus*) pre-stabilized at neutral pH (7.0). Fluorescence spectra were recorded, under the tested conditions, after contact using a spectrofluorometer. This procedure was designed to evaluate localized electronic perturbations of the N-doped surface functional groups resulting from direct adhesion to the bacterial cell walls. By comparing the response of the CD-emulgel to the smart-emulgel, the influence of the encapsulated REO on the initial bacterial attachment and signal transduction was distinguished from the effects associated with subsequent metabolically induced pH changes.

#### 4.6.5. Control Assessment of Intrinsic pH Sensitivity (Chemical Baseline)

As a chemical control, the intrinsic pH sensitivity of the CD-emulgel and smart-emulgel was evaluated in a sterile, cell-free environment. Fluorescence spectra were recorded over a broad pH range (2 to 12) using standardized buffer solutions. This analysis established a baseline for the protonation and deprotonation states of the N-doped surface functional groups in response to purely chemical stimuli. Comparing these baseline spectra with those obtained from the pathogen-laden samples allowed for the differentiation between signals derived from direct biological interactions and those triggered by the accumulation of acidic metabolic byproducts during bacterial proliferation.

The optical responsiveness of the QS-CDs and the resulting emulgel complexes to chemical stimuli was further evaluated at pH 2, 7, and 12, representing acidic, neutral, and alkaline environments, respectively. The pH of the aqueous dispersions and emulgel matrices was precisely adjusted using 0.1 M HCl and 0.1 M NaOH to simulate the chemical fluctuations associated with bacterial metabolism and environmental degradation. The dual-mode sensing capability was validated through synchronized colorimetric monitoring and fluorometric sensitivity analyses. For colorimetric monitoring (naked-eye detection), visible color changes were documented under ambient lighting conditions to assess the system’s potential as a real-time indicator for metabolic activity. The neutral condition (pH 7) was used as the reference state for comparison with the acidic (pH 2) and alkaline (pH 12) environments. This approach enabled the evaluation of the macroscopic color variations of the doped carbon framework in response to the acidification or alkalization of the surrounding medium. Simultaneously, fluorescence sensitivity was assessed by monitoring the pH-dependent quenching or enhancement of photoluminescence. These optical transitions were utilized to monitor changes in the protonation state of the nitrogen- and phosphorus-rich functional groups present on the QS-CDs surfaces. By correlating these shifts at specific pH conditions, the investigation aimed to link the microscopic electronic changes of the nanostructures with the specific metabolic signatures of the bacterial environment, thereby providing a robust platform for both visual and instrumental food freshness monitoring.

#### 4.6.6. Thermogravimetric Analysis (TGA/DTG) and Differential Scanning Calorimetry (DSC)

Thermogravimetric analysis (TGA) of the synthesized polymer powder was carried out using a Perkin Elmer thermogravimetric analyzer (Perkin Elmer, Waltham, MA, USA). Samples were heated from room temperature to 1000 °C at 10 °C/min under a nitrogen atmosphere. The thermal decomposition kinetics were subsequently analyzed using the Coats-Redfern equations. The degree of conversion (α) was calculated as

α = (W_0_ − W_t_) (7)

where W_0_ and W_t_, represent the initial, and instantaneous (at time t)weights, respectively.

The activation energy (E_a_) was determined using the following linearized equations:

(8) $$log\;\left[\frac{1-{\left(1-\alpha \right)}^{1-n}}{T^{2}\left(1-\alpha \right)}\right]=log\frac{AR}{\beta E}-\;\frac{E}{2.303RT}\;\;\;\mathrm{for}\;n\neq 1$$

(9) $$llog\;\left[\frac{-\log\left(1-\alpha \right)}{T^{2}}\right]=log\frac{AR}{\beta E}\left[1-\frac{2RT}{E}\right]-\frac{E}{2.303RT}\;\;\;\mathrm{for}\;n=1$$

The other kinetic parameters, including enthalpy (ΔH), entropy (ΔS) and free energy change (ΔG) were estimated as follows:

(10) ∆ H=E−RT

(11) ∆G=∆H−T∆S

(12) $$∆s=2.303\;\left(log\frac{Ah}{kT}\right)R$$

where E, h and k are the activation energy, Planck’s constant, and Boltzmann’s constant, respectively [54].

Differential scanning calorimetry (DSC) measurements were carried out using TA Instruments SDT Q600 V20.9 equipment. The specimens were heated to 600 °C at a rate of 10 °C/ min, using dry nitrogen as a purge gas. The relative degree of crystallization (X_c_) was estimated according to the following equation:

(13) $$Xc=\left(\frac{A_{\left(t\right)}}{A_{\left(total\right)}}\right)$$

where A(t) is the partial area of the peak at time t, and A(total) is the total peak area [55].

#### 4.6.7. Density Functional Theory (DFT) Calculations

DFT calculations were performed using the Gaussian 09W software package. To simulate the complex, high-molecular-weight biopolymer environment within a computationally feasible framework, simplified molecular fragments and localized clusters were utilized.

The raw cellulose backbone was represented by a cellobiose dimeric repeating unit featuring an intact β-(1,4)-glycosidic linkage, while the engineered amphiphilic CMC model was simulated by strategically grafting a carboxymethyl moiety onto the C6 position of the corresponding dimeric pyranose ring.

Concurrently, the nitrogen-doped QS-CDs were modeled as highly localized, nitrogen-functionalized polycyclic carbonaceous clusters terminated with amino (-{NH_2_) and carboxylic (-COOH) groups. These representative fragments were then geometrically optimized to their lowest ground-state energy conformations to accurately calculate the frontier molecular orbital eigenvalues, total energies, and dipole moments for both the individual components and their assembled emulgel complexes.

All molecular structures were optimized to their local minima using the Berny algorithm at the **B3LYP/6-31G(d,p)** level of theory. Following geometry optimization, key electronic parameters were extracted, including ground-state total energies (E_T_), frontier molecular orbital energies (E_HOMO_ (the highest occupied molecular orbital energy) and E_LUMO_ (the lowest unoccupied molecular orbital energy)), and the corresponding energy gap (E_g_). Furthermore, global reactivity descriptors such as dipole moment (µ), absolute hardness (η), absolute softness (S), and electrophilicity (ω) were determined to elucidate the molecular reactivity and the electrostatic zipping sensing mechanism of the smart-emulgel [34,44].

(14) $$E_{gap}=(E_{LUMO}-E_{HOMO})$$

(15) $$\eta =\frac{\left(E_{LUMO}-E_{HOMO}\right)}{2}$$

(16) $$\sigma =\frac{1}{\eta }$$

(17) $$S=\frac{1}{2\eta }$$

(18) $$\Delta N_{max}=\frac{-Pi}{\eta }$$

#### 4.6.8. Antimicrobial Activity via Optical Density (OD) Measurement

The antimicrobial activity of the treated samples was quantitatively assessed by monitoring the optical density (OD) at 600 nm. Fresh working stock cultures of the selected bacterial and fungal strains (*Escherichia coli* (ATCC 25922), and *Staphylococcus aureus* (ATCC 6538) were prepared by aseptically inoculating 5.0 mL of sterile nutrient broth (NB) with a loopful of microorganisms maintained on nutrient agar slants, followed by overnight incubation at 37 °C.

The inoculum concentration was adjusted to approximately 0.5 McFarland units, corresponding to a cell density of 1.5 × 10^8^ CFU/mL. For the assay, 25 µL of the standardized microbial suspensions was inoculated into 100 mL conical flasks containing 20 mL of sterile nutrient broth. The treated textile specimens were then introduced into the respective flasks and incubated at 37 °C for 24 h.

After incubation, the microbial growth was quantified by measuring the absorbance at 600 nm using a UV-Vis spectrophotometer. The relative antimicrobial efficiency was determined by calculating the percentage reduction in optical density (OD %) of the treated samples relative to the untreated control, according to the following equation:

(19) $$OD\left(\%\right)=\left(\frac{A-B}{A}\right)\times 100$$

where A represents the optical density of the control flask containing only the bacterial culture, and B the optical density of the flask containing the bacterial culture in the presence of the tested material.

#### 4.6.9. Visual Characterization and Phase Stability Imaging

The macroscopic phase behavior and colorimetric shifts of the emulgel formulations were systematically tracked and documented chronologically. To ensure reliable qualitative comparisons and minimize artificial background variations, all photographs were captured using a high-resolution digital camera phone (Samsung, Suwon, Republic of Korea) under standardized imaging conditions. Crucially, the sample tubes were positioned at the same location and photographed from a fixed perpendicular distance under constant ambient laboratory lighting throughout the observation period. All formulations were evaluated in identical glass tubes with the same internal diameters and equal sample depths to standardize the light path length. These precautions ensured that the documented structural developments, color variations, and phase boundaries reflected actual colloidal transitions rather than artifacts arising from variations in imaging conditions.

## Figures and Tables

**Table 1:** The quantum chemical QS-CDs, cellulose, CMC0.4, and REO oil.

DFT	QS-CDs	Cellulose	CMC	REO
**E_LUMO_ (eV)**	−0.0816	−3.4123	−3.3497	−1.1352
**E_HOMO_ (eV)**	−8.6232	−4.7538	−4.4898	−2.8155
**E_g_ (eV)**	8.5416	1.3415	1.1401	1.6802
**E_T_ (au)**	−581.27	−1208.88	−1434.55	−1068.82
**μ (Debye)**	1.76	12.63	11.14	2.66
**σ (eV)**	0.2341	1.4908	1.7541	1.1902
**ω (eV)**	2.2178	12.4277	13.4760	2.3223
**S (eV)**	0.1170	0.1170	0.8770	0.5951
**ɳ (eV)**	4.2708	0.6707	0.5700	0.8401

**Table 2:** The quantum chemical CMC/chitosan, REO-emulgel, CD-emulgel and smart-emulgel.

DFT	CMC/Chitosan	REO-Emulgel	CD-Emulgel	Smart Emulgel
**E_LUMO_ (eV)**	−2.5064	−3.3922	−4.3070	−3.6246
**E_HOMO_ (eV)**	−5.4724	−3.7042	−4.8460	−3.9783
**E_g_ (eV)**	2.9659	0.3121	0.5390	0.3537
**E_T_ (au)**	−2018.41	−3087.72	−2603.01	−3671.86
**μ (Debye)**	12.17	21.04	5.70	13.76
**σ (eV)**	0.6743	6.4078	3.7101	5.6537
**S (eV)**	0.3371	3.2039	1.8550	2.8268
**ɳ (eV)**	1.4830	0.1560	0.2695	0.1768

**Table 3:** TGA/DTG data for CD-emulgel and smart-emulgel.

Sample	Stage	TGA Range, °C	DTG Peak, °C	ML (%)	*n*	R^2^	∆G	∆H	∆s	A	SE	Ea
**CD-emulgel**	1st	48.23–208.21	118.75	23.09	1.0	0.9714	242.57	10.77	−0.24	3.88	59 × 10^−7^	18.69
**Smart-emulgel**	1st 2nd	42.00–182.03 600.06–720.03	125.43 679.60	59.67 8.06	1.5 1.5	0.9824 0.9995	112.67 316.22 **⅀G = 428.89**	18.83 87.27 **⅀H = 106.1**	−0.23 −0.24	4.12 5.56	57 × 10^−2^ 11 × 10^−2^	22.122 95.19

**Table 4:** DSC data for CD-emulgel and smart-emulgel.

Sample	T_m1_	T_m2_	T_exo,max_	X_c_
**CD emulgel**	92.79	137.06	572.11	0.11
**Smart emulgel**	92.13	132.13	579.48	0.14

## Data Availability

Data are available within the manuscript.
